# Comparative Metabolite Fingerprinting of Four Different Cinnamon Species Analyzed via UPLC–MS and GC–MS and Chemometric Tools

**DOI:** 10.3390/molecules27092935

**Published:** 2022-05-04

**Authors:** Mohamed A. Farag, Eman M. Kabbash, Ahmed Mediani, Stefanie Döll, Tuba Esatbeyoglu, Sherif M. Afifi

**Affiliations:** 1 Pharmacognosy Department, College of Pharmacy, Cairo University, Kasr El Aini St., Cairo 11562, Egypt; 2 Phytochemistry Department, National Organization for Drug Control and Research, Giza 12622, Egypt; emmy_700@hotmail.com; 3 Institute of Systems Biology (INBIOSIS), Universiti Kebangsaan Malaysia (UKM), Bangi 43600, Selangor, Malaysia; medianiahmed47@gmail.com; 4 German Centre for Integrative Biodiversity Research (iDiv) Halle-Jena-Leipzig, Puschstraße 4, 04103 Leipzig, Germany; stefanie.doell@idiv.de; 5 Institute of Biodiversity, Friedrich Schiller University Jena, Dornburger-Str. 159, 07743 Jena, Germany; 6 Department of Food Development and Food Quality, Institute of Food Science and Human Nutrition, Gottfried Wilhelm Leibniz University Hannover, Am KleinenFelde 30, 30167 Hannover, Germany; 7 Pharmacognosy Department, Faculty of Pharmacy, University of Sadat City, Sadat City 32897, Egypt

**Keywords:** chemometrics, *Cinnamomum*, erythritol, metabolomics, proanthocyanidin

## Abstract

The present study aimed to assess metabolites heterogeneity among four major *Cinnamomum* species, including true cinnamon (*Cinnamomum verum*) and less explored species (*C. cassia*, *C. iners*, and *C. tamala*). UPLC-MS led to the annotation of 74 secondary metabolites belonging to different classes, including phenolic acids, tannins, flavonoids, and lignans. A new proanthocyanidin was identified for the first time in *C. tamala,* along with several glycosylated flavonoid and dicarboxylic fatty acids reported for the first time in cinnamon. Multivariate data analyses revealed, for cinnamates, an abundance in *C. verum* versus procyandins, dihydro-coumaroylglycosides, and coumarin in *C. cassia*. A total of 51 primary metabolites were detected using GC-MS analysis encompassing different classes, *viz*. sugars, fatty acids, and sugar alcohols, with true cinnamon from Malaysia suggested as a good sugar source for diabetic patients. Glycerol in *C. tamala*, erythritol in *C. iners*, and glucose and fructose in *C. verum* from Malaysia were major metabolites contributing to the discrimination among species.

## 1. Introduction

Cinnamon is produced mainly from the dried inner bark of various evergreen trees from the genus *Cinnamomum* [1]. The genus *Cinnamomum*, a member of the Lauraceae family, includes ca. 250 species cultivated widely in sub-tropical and tropical Asia, Africa, and South America for their culinary and medicinal attributes [2]. As it is traded on a global scale, cinnamon also has economic importance, with Sri Lanka considered the world’s largest supplier of cinnamon products. It was reported that Sri Lanka exported cinnamon in various forms in 2016, with an estimated value of USD 167 million [3]. There are two distinct species of cinnamon, namely, *C. verum* (syn. *C. zeylanicum*), known as true cinnamon, and *C. cassia* (syn. *C*. *aromaticum*.), recognized as Chinese cinnamon [4]. True cinnamon, also known as Ceylon cinnamon, is indigenous to Sri Lanka [5], while Chinese cinnamon is native to South-East China [6]. True cinnamon has been substituted by Chinese cinnamon, available at a much lower price, albeit the latter encompassed higher levels of coumarin (ca. 0.31 mg/g), posing health risks when consumed regularly owing to its hepatotoxicity [7]. Other *Cinnamomum* species included *C. tamala* from North India [8] and *C. iners* from Central Malaysia [9].

Cinnamon is marked by a pungent aromatic taste with a spicy warm woody fragrance that is mediated mainly via its chemicals (*E*)-cinnamaldehyde and eugenol [10]. Therefore, cinnamon is employed as a seasoning and flavoring agent in cuisine, baked products, ice cream, and confections [11], but the presence of a toxic compound, coumarin, has raised safety concerns [7]. It was pointed out that (*E*)-cinnamaldehyde may constitute 40 to 90% of the volatile oils obtained from cinnamon bark [12]. During the storage of cinnamon oil, (*E*)-cinnamaldehyde is subjected to oxidation, forming benzaldehyde [13].

Aside from its culinary properties, cinnamon has been widely used in traditional folk medicine to relieve headache, toothache, flatulence, amenorrhea, common cold, and diarrhea [14]. Pharmacological studies confirmed cinnamon bark’s therapeutic effects *viz*., antimicrobial, anti-ulcerogenic, anti-allergic, antihypertensive, antioxidant, antidiabetic, antipyretic, hypolipidemic, and chemo-preventive effects [15]. A myriad of these biological activities, i.e., anti-obesity, cytotoxic, antibacterial, anti-mutagenic, anti-hyperglycemic, were attributed to (*E*)-cinnamaldehyde [16].

When considering its unique chemical composition, cinnamon is well-known for its richness in aromatics, diterpenes, and polyphenols [6]. Previous studies reported that *C. zeylanicum* comprised terpenoids, tannins, alkaloids, saponins, and considerable quantities of flavonoids and phenolics [17], while (*E*)-cinnamaldehyde, benzyl alcohol, and eugenol are the major components of the essential oil [18]. However, another study on the same species revealed that (*E*)-cinnamaldehyde, cinnamyl acetate, and linalool were the predominant constituents of the essential oil [19] and suggestive of origin based differences as typical in an essential oil [20]. Diethyl malonate (7%) was considered a major component besides (*E*)-cinnamaldehyde in *C. tamala* bark oil [21]. Differences in herbal product composition based on origin, genotype, or processing methods warrant for development of analytical methods for the quality control of these valuable drugs, as in the case of cinnamon [22].

Recently, advanced chemical profiling has made a tremendous contribution to the quality control and analysis of food metabolomics or foodomics [23,24]. In order to analyze food components on a metabolite level, hyphenated techniques such as liquid chromatography (LC) or gas chromatography (GC) coupled to mass spectrometry (MS) are performed, improving the opportunity to detect minor or novel metabolites in a complex sample [25]. Additionally, the quantitative NMR metabolomics approach was previously used to distinguish between true cinnamon (*C. verum*) and Chinese cinnamon (*C. cassia*), which are interchangeably used in food products [22].

Aside from its excellent resolution and high mass accuracy, high throughput UPLC-MS-based metabolomics represented key steps in investigating phytochemical differences across various plant genera in addition to closely related species and taxa [26]. With stable separation and great peak resolution, GC-MS is a powerful platform providing metabolite annotation in a relatively simple manner [27]. While most studies on cinnamon have targeted certain classes of metabolites, a large-scale metabolomics analysis was adopted in this study to characterize the secondary and primary constituents of this economically important spice in the context of the genetic diversity depicted by four different *Cinnamomum* species *viz*., *C. cassia*, *C. iners*, *C. tamala* and *C. verum*. This study provides detailed insight into the *Cinnamomum* species’ bioactive makeup to achieve reliable sample classifications in addition to biomarkers discovery of *C. verum* adulteration. A comprehensive profile for the main metabolites distinguishing *C. verum* based on UPLC-MS and GC-MS metabolomics approaches is presented and to be adopted for determining other factors or in other nutraceuticals.

## 2. Results and Discussion

### 2.1. Secondary Metabolites Profiling Using UPLC-ESI-MS

**Figure 1:**
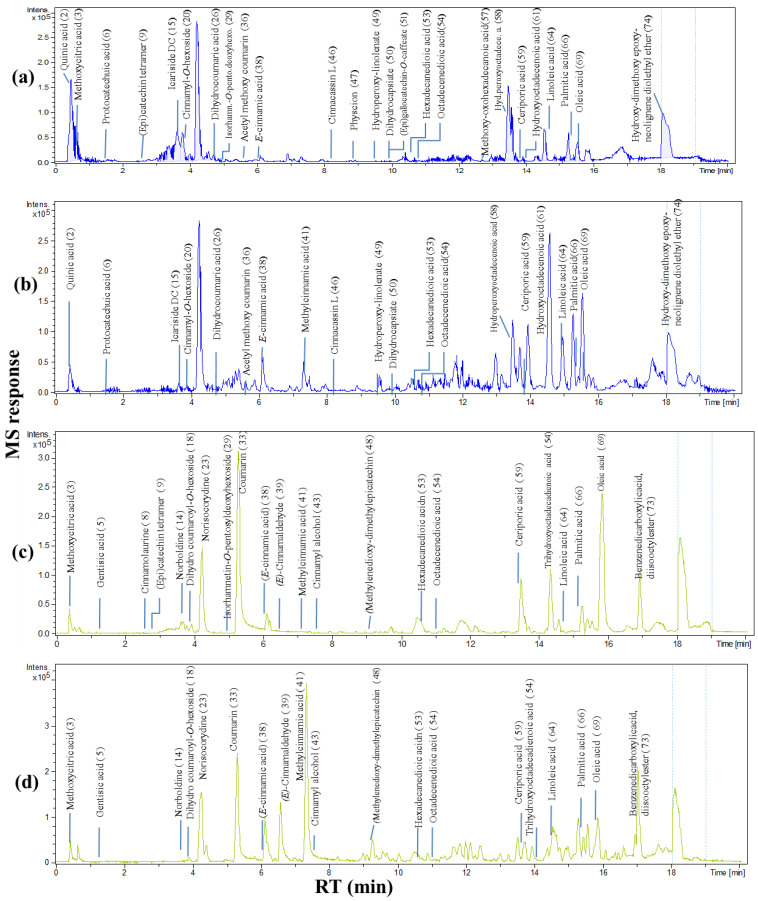
Representative Ultra -Performance Liquid Chromatography–Mass Spectrometry (UPLC-MS) base peak chromatogram of cinnamon bark 70% methanol extract in negative ion mode (**a**) CC (*Cinnamomum cassia* from Malaysia), (**b**) CV (*C. verum* from Pakistan), and positive ion mode (**c**) CC, (**d**) CV.

UPLC-ESI-MS analysis was carried out in both negative and positive ionization modes, allowing the annotation of 74 metabolites (Figure 1) in the examined four *Cinnamomum* species from which *C. verum* was obtained from two different sources, in addition to *C. cassia*, *C. iners*, and *C. tamala*. Metabolites belonged to different classes, including phenolic acids, tannins, phenyl propanoids (i.e., hydroxycinnamates, coumaroyl derivatives), flavonoids, lignans, amides, terpenes, and fatty acids (i.e., dicarboxylated and tricarboxylated fatty acids). A list of identified peaks along with their chromatographic and spectroscopic data is presented in Table 1. The structures and fragmentation patterns of some identified metabolites discussed in the manuscript are shown in the Supplementary Material (Supplementary Figures S1–S7). Metabolites were eluted based on their polarity in descending order. Positive and negative ionization modes provide greater coverage of the metabolome. The negative ionization mode showed better sensitivity than the positive mode, with lower noise and higher signal-to-noise ratios except for a few compounds (ca. 21 peaks), including alkaloids and some hydroxycinnamates, which readily ionized in the positive ion mode.

#### 2.1.1. Proanthocyanidins

Proanthocyanidins show UV-absorbance maxima at 235 and 280 nm corresponding to the absorbance spectrum of flavan-3-ols. UPLC-ESI-MS^n^ led to the identification of five procyanidins (PAs) and two prodelphinidins with a strong deprotonated molecular ion [M − H]^−^ in the negative ion mode. Common fragmentation pathways for PAs include Retro Diels–Alder fission (RDA-F) [M − H-152]^−^, heterocyclic ring fission (HRF) [M − H-126]^−^, benzofuran-forming fission (BFF) [M − H-122]^−^, and quinine methide cleavage (QMC) that yielded characteristic ions for sequencing of PAs [61].

##### Procyanidin

As depicted from the UPLC chromatograms in Figure 1, two A-type (epi)catechin tetramers (peaks L9, L10) and three trimers (peaks L11, L12, L25) procyanidins were identified in the studied extracts. Peak L9 showed deprotonated and protonated molecular ions at [(M − H)^−^ at *m*/*z* 1151.2454 (C_60_H_47_O_24_)^−^] and [(M + H)^+^ at 1153.2626 (C_60_H_49_O_24_)^+^] respectively, indicate a procyanidin trimer with A-type interflavanyl bonds. It had main fragment ions at *m*/*z* 863 [−288 amu], attributed to the loss of the upper (epi)catechin unit, and *m*/*z* 575 [M − H-(2 × 288)]^−^, indicating the presence of the A-type bond between the third and the terminal flavan-3-ols units (Supplementary Figure S1) and annotated as (epi)catechin tetramer. It was identified in *C. cassia* and *C. iners* while being absent in other specimens. Peak L10 [(M − H)^−^ at *m*/*z* 1151.25, (C_60_H_47_O_24_)^−^] showed MS^2^ fragments at *m*/*z* 863 [−288 amu] due to the loss of upper EC unit and *m*/*z* 573 due to the successive loss of terminal EC unit (Supplementary Figure S2), indicative for the presence of the A-type bond between the second and third flavan-3-ol. This tetramer was found only in *C. cassia* species. Therefore, peak L10 could serve as a marker for that species among cinnamon food products has yet to be confirmed.

Peak L11 [(M − H)^−^ at 863.1885 (C_45_H_35_O_18_)^−^] showed fragment ion peaks at *m*/*z* 711 [M − H-152] due to RDA, and *m*/*z* 573 [M − H-290] related to the loss of terminal (epi)catechin suggesting for the presence of A linkage between the top and middle units (Supplementary Figure S3A). While main fragments in peak L12 [(M − H)^−^ at 863.1853 (C_45_H_35_O_18_)^−^] were at *m*/*z* 577 [M − H-286] (loss of extension A-type (E)C unit), *m*/*z* 427 (loss of extension (E)C unit and RDA fragmentation) and *m*/*z* 289 related to the terminal (E)C unit (Supplementary Figure S3B). This may indicate that the structural differences in L11 and L12 lie in the stereochemistry of their subunits (epicatechin/catechin) and thus cannot be distinguished by means of MS and with both annotated as A-type (epi)catechin trimer C. Peak L25 with deprotonated and protonated molecular ion peaks [(M − H)^−^ at *m*/*z* 861.1699 (C_45_H_33_O_18_)^−^] and [(M + H)^+^ at *m*/*z* 863.1811 (C_45_H_35_O_18_)^+^], respectively, suggested for a procyanidin trimer with two A-type interflavonoid linkages. It was detected only in *C. cassia* and *C. tamala.* The MS^2^ fragment ions at *m*/*z* 575 [M − H-286]^−^ are due to the loss of upper EC unit by QM cleavage and consequently annotated as (epi)catechin trimer with double A linkage. It was noticed that no PA trimers were detected in both *C. verum* accessions from either origin (Pakistan or Malaysia). This may account for its less astringent taste than *C. cassia* based on its tannins content [22].

##### Prodelphinidins

Prodelphinidin detection is particularly interesting as the pyrogallol group in gallo(epi)catechins (EG) is related to the biological activity of grape and tea polyphenols, as previously reported [32]. Thus, the identification of these substructures may explain some of the properties of cinnamon extracts. Among the studied extracts, it was detected only in *C. tamala*; thus, it may give this species more attention regarding its biological activity.

The main fragments detected in peak L51 [(M − H)^−^ 467.097 (C_24_H_19_O_10_)^−^] were at *m*/*z* 313 (due to RDA fragmentation) and *m*/*z* 161 (due to loss of terminal (E)G unit) annotated as epigallocatechin-*O*-caffeate. It was previously detected in tea extract, albeit it is the first time that it has been detected in cinnamon. The pyrogallol moieties of (epi)gallocatechins (e.g., in tea) are more reactive than the catechol moieties of (epi)catechins regarding their antioxidant activity [62]. Peak L52 [(M − H) 593.2696 (C_30_H_41_O_12_)^−^] showed fragment ions at *m*/*z* 467 [M − H-126] due to HRF and *m*/*z* 305 due to QM cleavage between EC and EG units assigning this peak as gallo(epi)catechin-(epi)catechin.

A novel procyanidin was detected in peak L37 [(M − H)^−^ 995.2404 (C_51_ H_47_ O_21_)^−^] (Supplementary Figure S4) in *C. tamala* and absent in other specimens. It exhibited MS^2^ fragment ions at *m*/*z* 705 [M − H-290]^−^ due to the loss of terminal EC, *m*/*z* 543 [M − H-290-162]^−^ and *m*/*z* 289 [M − H-290-162-254]^−^ due to the loss of hexose and chrysin moieties. The fragmentation pattern suggested its annotation as a new procyanidin containing two (epi)catechin units attached to chrysin and a hexose moiety. Further investigation is required to confirm this identification using other spectroscopic tools.

#### 2.1.2. Hydroxycinnamates (HCAs)

Hydroxycinnamates (HCAs) represent one of the characteristic constituents in cinnamon, which possess various biological activities such as antitumor, anti-inflammatory, antioxidant, and neuroprotective activities [63]. Eight cinnamyl derivatives (peaks L20, L24, L27, L38, L39, L42, L43, and L63) were identified in both negative and positive ionization modes. Peak L38 showed (M − H)^−^ [*m*/*z* 147.0447 (C_9_H_7_O_2_)^−^] with the main fragment ion at *m*/*z* 103 [(M − H-44)^−^], identified as cinnamic acid, and more prominent in *C. verum* from Malaysia and Pakistan. Cinnamic acid exhibited potential antibacterial activity [64], in addition to its anti-inflammatory and analgesic effect [65], posing these species to be further investigated for such indications. Peaks L20, L24, L27, and L62 were cinnamoyl glycosides showing characteristic sugar losses. Peak (L20) [(M − H)^−^ *m*/*z* 295.1155 (C_15_H_19_O_6_)^−^] was identified as cinnamyl-*O*-hexoside. Peak L24 [(M − H)^−^ *m*/*z* 427.1673 (C_20_H_27_O_10_)^−^] was identified as cinnamyl-*O*-pentosylhexoside (rosavin), previously reported in *C. cassia* [40], confirmed from fragment ion at *m*/*z* 293 [M − H-134]^−^ due to the loss of cinnamyl alcohol, while the fragment ions at *m*/*z* 233 and 191 appeared due to sugar losses. Herein It was detected only in *C. cassia*; thus, it may be used as a marker for this species. Peak L27 [(M − H)^−^ *m*/*z* 429.1762 (C_20_H_29_O_10_)^−^] with a mass difference of 2 amu compared to compound L24 was identified as dihydrocinnamyl-*O*-pentosylhexoside, which is the first time being reported in cinnamon (Supplementary Figure S5), and only identified in *C. iners* species. Furthermore, peak (L63) [(M − H)^−^ *m*/*z* 395.2781 (C_23_H_39_O_5_)^−^], with its fragment ion at *m*/*z* 263 [M − H-132]^−^, was identified as cinnamoylcinnamate-*O*-pentoside detected only in *C. iners* species. Whether peaks L27 and L63 could serve as markers for that species has yet to be confirmed. Peak L39 [(M + H)^+^ *m*/*z* 133.0652 (C_9_H_9_O)^+^] was identified as (*E*)-cinnamaldehyde that was reported to exhibit antibacterial, antifungal [66], antioxidant, and anti-inflammatory activities [67], in addition to its flavor imparting properties in cinnamon spice found most abundant in *C. verum* and *C. iners* compared to other species and to likely account for their pungent taste. Peak L42 [(M + H)^+^ *m*/*z* 163.076 (C_10_H_11_O_2_)^+^] was identified as methoxy-cinnamaldehyde, found most prominent in *C. verum* species. *C. verum* was reported to exhibit antitumor activity due to its richness in methoxy-cinnamaldehyde [68] and rationalizing for its superiority among cinnamon drugs. Standardization of methoxy-cinnamaldehyde and cinnamaldehyde should provide a better indication of cinnamon’s health benefits. Peak L43 [(M + H)^+^ *m*/*z* 135.081 (C_9_H_11_O)^+^] was identified as cinnamyl alcohol that was previously detected in bark and twigs of *C. cassia* [69].

Compounds L16, L17, L18, L19, and L33 were coumaroyl derivatives, a precursor to cinnamates. Peaks L18 and L19 showed the same molecular ions at *m*/*z* 327, whereas MS^2^ fragment ions revealed the main difference in their structures. Peak L18 showed fragment ion at *m*/*z* 283 [M − H-44]^−^, followed by *m*/*z* 165 [M − H-162]^−^, suggestive for the presence of free carboxylic group and annotated as dihydrocoumaroyl-*O*-hexoside and was identified in *C. cassia*, *C. tamala* and *C. verum* from Pakistan, while peak L19 showed fragment ion at *m*/*z* 165 [M − H-162]^−^, followed by *m*/*z* 121 [M − H-162-44]^−^, assigning it as dihydrocinnacasside. Likewise, peaks L16 and L17 showed the same (M − H)^−^ at *m*/*z* 459 with a mass difference of 132 amu than peaks L18 and L19, and annotated as dihydrocinnacasside-*O*-pentoside and dihydrocoumaroyl-*O*-pentosylhexoside, respectively. Dihydrocinnacasside-*O*-pentoside was identified in all *Cinnamomum* species except *C. cassia*. Peak L33 [(M + H)^+^*m*/*z* 147.0446 (C_9_H_6_O_2_)^+^] was identified as coumarin found most abundant in *C. cassia*, consistent with previous results ensuring its richness in coumarin with though health risks when consumed regularly. The lowest level of coumarin among the studied species was found in *C. iners*. This finding, together with the high level of cinnamaldehyde, poses its use as a flavoring agent instead of the more expensive true cinnamon.

#### 2.1.3. Flavonoids

Three flavonoid glycosides were detected for the first time in *Cinnamomum* species, including peak L28 [(M − H)^−^ *m*/*z* 595.1699 (C_27_H_29_O_17_)^−^] and peak L29 [(M − H)^−^ *m*/*z* 593.1848 (C_28_H_33_O_14_)^−^] assigned as naringenin di-*O*-hexoside in *C. iners* and *C. tamala* and isorhamnetin-*O*-pentosyldeoxyhexoside in *C. cassia* and *C. tamala*, respectively. A novel *O*- and *C*-glycosylated flavonoid (peak L30) was detected for the first time in *C. cassia* and *C. tamala*. Peak L30 [(M − H)^−^ *m*/*z* 609.1998 (C_27_H_29_O_16_)^−^] showed main fragment ions at *m*/*z* 447 [M − H-162]^−^ (due to loss of *O*-linked hexose sugar), and *m*/*z* 327 [M − H-162-120]^−^, indicating the presence of *C*-linked hexose moiety and annotated as luteolin-*O*-hexosyl-*C*- hexoside (Supplementary Figure S6). Peak L28 showed main fragment ions at *m*/*z* 433 [M − H-162]^−^ and *m*/*z* 271 [M − H-162 × 2]^−^ confirming naringenin as its aglycone, whereas peak L29 showed main fragment ions at *m*/*z* 447 [M − H-146]^−^ and *m*/*z* 315 [M − H-146-132]^−^ for isorhamnetinaglycone.

#### 2.1.4. Alkaloids

UPLC-MS/MS analysis in positive ionization mode identified several alkaloids belonging to the isoquinoline type—mainly benzylisoquinolines and aporphines [70]. Peak L14 [(M + H)^+^*m*/*z* 314.1382 (C_18_H_20_NO_4_)^+^] showed a fragment ion at *m*/*z* 297 [M + H-17]^+^ and was identified as norboldine. Antiplasmodial and antiviral activities of norboldine were reported [70], with the relative highest levels in *C. iners* species, posing its extract to be tested for these effects in the future. Compared to peak L14 showing UV maxima at 220, 280, and 310 nm, peak L21 showed absorbance at 270 and 310 nm, suggested of a substituted aporphine. Its mass spectrum (Supplementary Figure S7) showed (M + H)^+^ at *m*/*z* 342.1678 (C_20_H_24_NO_4_)^+^] and a fragment ion peak at *m*/*z* 297 due to the opening of ring B and loss of methylene imine group as typical of aporphines and identified as corydine [39]. Peak L23 [(M + H)^+^ *m*/*z* 328.1528 (C_19_H_22_NO_4_)^+^] showed an ultraviolet absorption spectrum similar to that of peak L21 and was identified as norisocorydine. Peak L22 [(M + H)^+^ *m*/*z* 330.1680 (C_19_H_20_NO_4_)^+^] showed UV max typical of benzylisoquinolines annotated as reticuline, previously isolated from *C. camphora* [35].

#### 2.1.5. Lignans and Terpenes

Lignans from different plant sources were reported to show neuroprotective activities being useful in the treatment and prevention of neurodegenerative diseases [71]. In the present study, the negative ionization mode allowed the detection of two lignans, namely dihydroxy-tetramethoxy-epoxylignanol-7-one (peak L34 [(M − H)^−^ at *m*/*z* 433.1479 (C_22_H_25_O_9_^−^)]), cinnacassin L (peak L46 [(M − H)^−^ at *m*/*z* 281.1168 (C_17_H_18_O_3_^−^)]), and one neolignan; and hydroxy-dimethoxyepoxy-neolignenediolethyl ether (peak L74 [(M − H)^−^ at *m*/*z* 385.1657 (C_22_H_26_O_6_^−^)]), which were previously detected in the twigs of *C. cassia* [51]. The three compounds were detected in *C. iners* and *C. verum* from Pakistan. Different diterpenoids were previously detected in *C. cassia* with immunosuppressive activities that may play roles in the treatment or prevention of autoimmune diseases and chronic inflammatory disorders [59]. Herein two diterpenes were identified in all *Cinnamomum* species, namely cinncassiol B [(M − H)^−^ at *m*/*z* 399.1961 (C_20_H_31_O_8_^−^)] and cinncassiol A [(M − H)^−^ at *m*/*z* 381.1724 (C_20_H_29_O_7_^−^)].

#### 2.1.6. Fatty Acids

Negative ionization mode identified a number of saturated and unsaturated fatty acids, showing typical loss of H_2_O [M − H-18]^−^ and/or loss of carboxylic moieties [M − H-44]^−^. Peaks L64, L66, and L69 with [M − H]^−^ *m*/*z* 279.232 (C_18_H_31_O_2_)^−^, 255.2328 (C_16_H_31_O_2_)^−^, and *m*/*z* 281.2483 (C_18_H_33_O_2_)^−^, respectively, constituted the major peaks in all specimen, especially *C. verum* from both origins and *C. iners*. They were tentatively identified as linoleic acid, palmitic acid, and oleic acid, respectively. A mass difference of 16 amu between peaks L64 and L55 was indicative of an extra hydroxy group and assigning peak L55 as hydroxy linoleic acid identified in *C. tamala* and *C. verum* of both origins. Likewise, peak L60, a trihydroxylated fatty acid, was detected in cinnamon for the first time as trihydroxyoctadecaenoic acid [(M − H)^−^, *m*/*z* 327.2167] with the main fragment ion at *m*/*z* 283 due to the loss of carboxylic group. It was detected in *C. iners* and *C. verum* of both origins. Three dicarboxylic fatty acids were observed for the first time in cinnamon annotated as hexadecanedioic acid (L53) [*m*/*z* 285.2060, (C_16_H_30_O_4_)^−^] in *C. iners* and *C. verum* of both origins, octadecenedioic acid (L54) [*m*/*z* 311.2205, (C_18_H_31_O_4_)^−^] in all *Cinnamomum* species, and hexadecanedioic acid methyl ester (L57) [*m*/*z* 299.2060, (C_17_H_31_O_4_)^−^] in all *Cinnamomum* species except *C. verum* (Supplementary Figure S8A–C).

### 2.2. Multivariate Data Analysis of UPLC-ESI-MS Data

**Figure 2:**
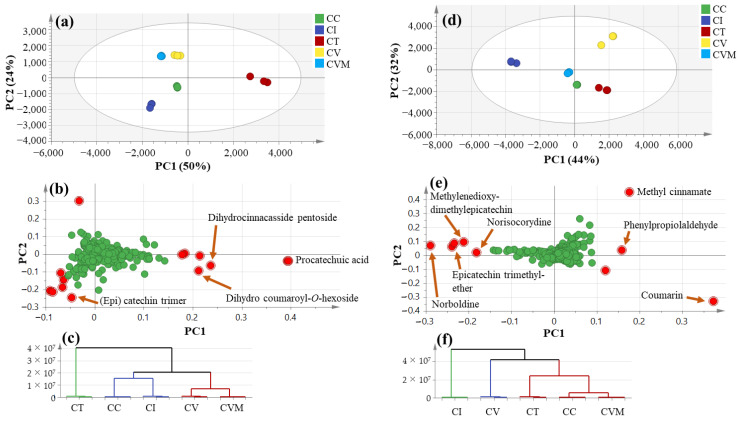
UPLC-MS principal component analyses of the different cinnamontaxa (*n* = 3) on negative ion mode: (**a**) Score plot of PC1 vs. PC2, (**b**) respective loading plot with contributing mass peaks, (**c**) HCA and on positive ion mode, (**d**) Score plot of PC1 vs. PC2, (**e**) respective loading plot, (**f**) HCA. CC: *Cinnamomum cassia* from Malaysia, CI: *C. iners* from Malaysia, CT: *C. tamala* from Pakistan, CV: *C. verum* from Pakistan, CVM*: C. verum* from Malaysia.

Although the visual inspection of the UPLC-MS chromatograms (Figure 1) of the examined species revealed different metabolite patterns. The data were further analyzed in a more holistic way using principal component analysis (PCA) to assess the variance within specimens in an untargeted manner. PCA is an unsupervised multivariate data analysis technique requiring no knowledge of the data set and was used to explain metabolite differences and possible discrimination between the studied species [72]. The PCA model for the studied species in negative mode (Figure 2a–c) accounted for 50% of the total variance in the first component, PC1, whereas the second principal component, PC2, explained 24% of the variance. The score plot (Figure 2a) revealed the clustering of the two true *Cinnamomum* specimens *C. verum* (CV from Pakistan and CVM from Malaysia) from different locations together in one quadrant, while *C. iners* (CI) and *C. cassia* (CC) were at another quadrant with negative PC1 values. On the other hand, *C. tamala* (CT) was separated from the rest of the samples with positive PC1 values. The loading plot (Figure 2b) revealed that phenolic acids, i.e., protocatechuic acid, dihydrocinnacasside pentoside and dihydro coumaroylhexoside were responsible for the segregation of CT in a separate quadrant from the rest of the other species. Hierarchical clustering analysis (HCA) (Figure 2c) confirmed the same clustering pattern obtained from the PCA model.

The UPLC-MS dataset from the positive ionization mode was also subjected to PCA analysis (Figure 2d–f), showing relatively different clustering for the studied cultivars with PC1 and PC2 to account for 44 and 32%, respectively. The score plot (Figure 2d) showed likewise that CI was the most segregated species among specimens, whereas CV failed to cluster with CVM as they represent the same species and opposite to negative ion mode results (Figure 2a). The rest of the cinnamon specimens were clustered together. The loading plot (Figure 2e) showed that CI was more enriched in (epi)catechins represented by methylenedioxy-dimethylepicatechin and epicatechintrimethylether, as well as alkaloids represented by norboldine and norisocorydine, and warrant for the profiling of plant extracts in different ionization modes. In contrast, methyl cinnamate and coumarin were most abundant in CV and CT, respectively. To identify whether variant metabolites revealed from PCA could serve as potential markers for examined species, supervised partial least squares-discriminant analysis (OPLS-DA) was employed.

The data from negative ion mode were first subjected to OPLS-DA analysis (Supplementary Figure S9a,b) using *C. tamala* (CT) as first-class against all other species to identify the markers that are most distant among examined cinnamon specimens. OPLS-DA, as a supervised multivariate data analysis technique, has greater potential in the identification of markers by providing the most relevant variables for the differentiation between two class groups. OPLS results confirmed PCA regarding the richness of *C. tamala* in protocatechuic acid at a *p*-value less than 0.05. In the positive ionization mode, another OPLS-DA model (Supplementary Figure S9c,d) using CI against other samples confirmed its richness in (epi)catechins and alkaloids concurrent with lower levels of coumarin. These findings rank CI as the closest species to CV, suggesting the former as a potential substitute for true cinnamon with minimal coumarin health risk. In an attempt to distinguish between true (CV and CVM) and Chinese cinnamon (CC), especially since CC is the common adulterant of true cinnamon, the OPLS-DA model was conducted in the negative (Supplementary Figure S10a,b) and positive ionization (Supplementary Figure S10c,d) modes. Supplementary Figure S10 revealed that (epi)catechin trimer A type, dihydrocoumaroylhexoside, dihydrocoumaroyl-*O*-pentosylhexoside, and coumarin were characteristic markers for Chinese cinnamon. Novel markers for true cinnamon revealed in this study included methyl cinnamate and cinnamoyl alcohol and are suggestive of the abundance of cinnamates in true cinnamon.

Lastly, to distinguish between true cinnamon of different origins that are CV and CVM, both specimens were modeled against each other using OPLS-DA (Figure S11a) in negative ion mode with R2 and Q2 values of 0.97 and 0.96, respectively. The S-loading plot (Supplementary Figure S11b) showed the exact markers for true cinnamon from Malaysia belonged mostly to phenolic acids, i.e., Protocatechualdehyde, cinnamic acid and protocatechuic acid. The most discriminatory metabolites, as revealed from UPLC-ESI-MS and multivariate analysis, were then subjected to ANOVA analysis to confirm their statistical significance in differentiating between the samples under study (Supplementary Table S2). *C. iners* showed a significantly higher level (*p* < 0.05) of (*E*)-Cinnamaldehyde and norboldine with the lowest level of coumarin. A significantly higher level (*p* < 0.05) of protocatechuic acid was observed in *C. tamala*, while true cinnamon showed its richness in cinnamate, including cinnamic acid, (*E*)-cinnamaldehyde, and methylcinnamic confirmed the results obtained from MVA.

### 2.3. Primary Metabolites Profiling Using GC-MS

Primary metabolites of cinnamon were assessed post-silylation using GC-MS analysis (Supplementary Figure S12) in order to account for the nutritive value of cinnamon. The results (Table 2) revealed 51 primary metabolites categorized into 10 various chemical classes, i.e., sugars, esters, amino acids, phenolics, organic and fatty acids. All *Cinnamomum* species were enriched in sugars and esters, while amino acids were present at much lower levels.

#### 2.3.1. Sugars

Sugars were the most abundant class in all *Cinnamomum* species except for CV (*C. verum* from Pakistan). The highest relative levels of total sugars were detected in CVM (*C. verum* from Malaysia) at ca. 64%, followed by CT at 53%. Monosaccharides were present at much higher levels compared to disaccharides represented by psicose (peaks G29, G30), glucose (G32, G33), and fructose (G31). Glucose (G32, G33) was the most dominant monosaccharide amounting to 23% in CVM versus the lowest levels in CI (*C. iners*) at ca. 2%. Psicose (G29, G30), a low-calorie monosaccharide sugar that is 70% as sweet as sucrose with anti-obesity and antidiabetic effects [73], was detected at the highest levels in true cinnamon from Malaysia, posing it as a good sugar source for diabetic patients. The only identified disaccharide sucrose (G34) was detected at 2% in CC (Chinese cinnamon) ca. three-folds higher than other cinnamon samples.

CI and CT contained the highest levels of sugar alcohols at 29 and 19%, respectively, versus the lowest levels (5%) in CVM (*C. verum* from Malaysia). Generally recognized as safe food additives, sugar alcohols are low digestible carbohydrates [74] and pose CI as a good source of sugar alcohols. Glycerol (G41) was the most abundant sugar alcohol in all *Cinnamomum* species except for CI (*C. iners*), where *meso*-erythritol (G43) and arabitol (G44, G45) were the major sugar alcohols detected at 16 and 6%, respectively. Among all sugar alcohols, *meso*-erythritol (43) and arabitol (G44, G45) provide the lowest calories (0.2 kcal/g) [75], posing CI as a potential low-calorie sweetener. Sensory analysis to compare taste preferences for CI compared to true cinnamon should now follow. As they possess antimicrobial activity [76], sugar acids were most enriched in *C. verum* from Malaysia (12%), while the lowest levels were detected in the same species from Pakistan (2%), suggesting geographical origin impact. However, such a hypothesis should be confirmed by analyzing true cinnamon samples from other origins. Major sugar acids detected in CVM included galactonic acid γ-lactone (G37, G38) at 12% of total metabolites composition.

#### 2.3.2. Fatty Acids/Esters

Fatty acyl esters constituted the second major class in all *Cinnamomum* species (16–34%), reaching the highest content in CV. Major fatty acid esters included glycerol monostearate (G4) and followed by 1-monopalmitin (G3) in all *Cinnamomum* species. Glycerol monostearate (G4) is broadly used in bakery products to enhance the taste and appearance of flour foods owing to its anti-staling properties that rationalize the incorporation of cinnamon in pastry aside from its role as a natural flavor [77]. Monoglycerides generally act as emulsifiers resulting in a more stable air dispersed baked cake with a relatively soft crumb [78]. The abundance of esters in *Cinnamomum* species was affected by the levels of their corresponding fatty acids.

Fatty acids were present in all *Cinnamomum* species at considerable levels reaching 12% in CV and accounting for its fatty taste [79]. Stearic (G13) and palmitic (G9) acids were the main fatty acids at ca. 4%. Subsequently, these saturated fatty acids act as precursors for their counterpart major esters in cinnamon. CVM (*C. verum* from Malaysia) contained the least free fatty acids level [80].

#### 2.3.3. Organic Acids

Another primary metabolite class posing quantitative differences among examined cinnamon specimens was organic acids to impart a slightly bitter taste, especially in CT (*C. tamala*), which has the highest levels (8%). Oxalic (G18), (*E*)-cinnamic (G23), and quinic (G25) acids were the major constituents of this class. Oxalic acid (G18) is considered an anti-nutrient, whereas quinic acid (G25) exhibits anti-inflammatory and immune-enhancing activities [81]. As it was detected at a seven-fold higher level in CVM than CV, (*E*)-cinnamic acid (G23) has a honey-like odor with anti-obesity effects [82]. The elevated levels of (*E*)-cinnamic acid (G23) in CVM (*C. verum* from Malaysia) compared to CV (*C. verum* from Pakistan) were in agreement with UPLC-MS results in negative ion mode (Supplementary Figure S11). Moreover, a direct correlation was observed between (*E*)-cinnamaldehyde and its precursor (*E*)-cinnamic acid, which is more enriched in CVM than CV.

#### 2.3.4. Phenolics

Phenolics were more abundant in CT (5%) and CV (2%) than in other cinnamon samples, and they are considered natural antioxidants [83]. Major phenolics detected using GC-MS included catechin (G27) and protocatechuic acid (G26) in all *Cinnamomum* species. Catechin (G27), a predominant component in tea, exhibited a bitter taste [84], though with many health benefits, including antioxidant and antidiabetic activities [85] contributing to the overall biological effects of cinnamon. Protocatechuic acid (G26) was present at much higher levels in CT at 5% as indicated by OPLS-DA-UPLC-MS results (Figure 2b) and in accordance with GC-MS results posing this accession as a potential antioxidant.

### 2.4. Multivariate Data Analysis Using GC-MS Data

**Figure 3:**
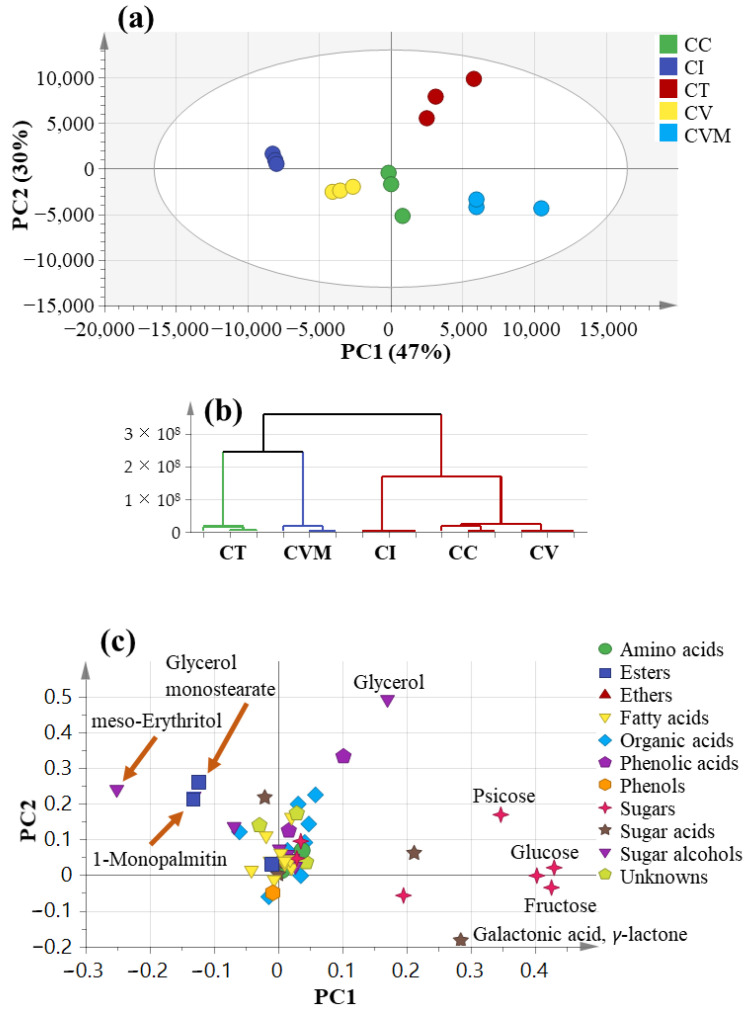
GC-MS principal component analyses of the different cinnamon taxa (*n* = 3) (**a**) score plot of PC1 vs. PC2, (**b**) respective loading plot with contributing chemical classes, and (**c**) HCA plot. The metabolome clusters are placed in two-dimensional space at the distinct locations defined by two vectors of principal component PC1 = 47% and PC2 = 30%. CC: *Cinnamomum cassia* from Malaysia, CI: *C. iners* from Malaysia, CT: *C. tamala* from Pakistan, CV: *C. verum* from Pakistan, CVM*: C. verum* from Malaysia.

The GC-MS data were likewise analyzed using PCA (Figure 3) to assess the variance within cinnamon specimens in an untargeted manner and to compare the classification potential of GC-MS compared to the UPLC-MS platform. The PCA model for the studied species (Figure 3a) explained 47% of the total variance in PC1, whereas the second principal component, PC2, explained 30% of the variance. HCA (Figure 3b) showed that CT was the most distant among other samples in agreement with UPLC-MS results (Figure 2a). However, this model failed to cluster CV and CVM together, which are of the same genotype and appear together from the UPLC-MS-based PCA model (Figure 2a). Examination of the loadings plot (Figure 3c) pointed out that glycerol (G41) and protocatechuic acid (G26) were more abundant in CT. Moreover, Cl (*C. iners*) was more enriched in sugar alcohols represented by *meso*-erythritol (G43), while CV (*C. verum* from Pakistan) encompassed more fatty acyl esters, i.e., glycerol monostearate (G4) and 1-monopalmitin (G3). On the right side of the loading plot along PC1, CVM (*C. verum* from Malaysia) was characterized by higher levels of sugar acids *viz*. galactonic acid *γ*-lactone (G37) and its isomer (G38) in addition to sugars *viz*., fructopyranose (G31), psicofuranose (G29/G30), and glucopyranose (32/33). Sugars are of low taxonomic value and are thus not clear markers for distinguishing CV and CVM accessions, which are dependent on agricultural practices or growing conditions [86].

Considering CT distant segregation in the PCA model, it was modeled as one class using OPLS-DA analysis (Supplementary Figure S13) versus all other species in order to identify its significant markers at a *p*-value less than 0.001. The OPLS-DA score plot (Supplementary Figure S13a) displayed model parameters R2 (goodness of fit) and Q2 (goodness of prediction) at 0.90 and 0.83, respectively, proving the good model predictability and fitness. The OPLS-DA derived S-plot (Supplementary Figure S13b) illustrated that protocatechuic acid (G26) was a marker for CT confirming results derived from UPLC-MS analysis (Figure 2b) in addition to glycerol (G41). Another OPLS-DA model (Supplementary Figure S14) was employed for identifying markers for Chinese cinnamon CC adulteration in true cinnamon CV with a *p*-value less than 0.001. The OPLS-DA score plot (Supplementary Figure S14a) also displayed good model prediction parameters R2 and Q2 at 0.99 and 0.91, respectively. The OPLS-DA derived S-plot (Supplementary Figure S14b) revealed sugars enrichment, i.e., glucopyranose (G32), fructopyranose (G31), and psicofuranose (G30) in CC (*Cinnamomum cassia*; Chinese cinnamon) compared to true cinnamon that displayed no specific markers.

## 3. Materials and Methods

### 3.1. Plant Material

Bark specimens of four different *Cinnamomum* species *viz*., *C. cassia*, *C. iners*, *C. tamala,* and *C. verum* were obtained from different sources with sample information presented in Supplementary Table S1. The bark from each specimen was separately homogenized with a mortar and pestle under liquid nitrogen and then stored in tight glass containers at −20 °C until further analysis. Vouchers of cinnamon specimens are deposited at the College of Pharmacy Herbarium, Cairo University, Egypt.

### 3.2. Chemicals

Formic acid and acetonitrile (HPLC grade) were provided by Baker (The Netherlands). All other solvents, standards, and chemicals were obtained from Sigma Aldrich (St. Louis, MO, USA).

### 3.3. UPLC-ESI-QTOF-MS Analysis and Metabolites Identification

Dried finely pulverized cinnamon specimens (10 mg) were extracted by adding 2 mL 70% MeOH, containing 10 μg mL ^−1^ umbelliferone as an internal standard sonicated for 20 min with frequent shaking, then centrifuged at 12,000× *g* for 10 min to remove debris. The filtered extract through a 0.22 μm filter was subjected to solid-phase extraction using a C_18_ cartridge (Sep-Pack, Waters, Milford, MA, USA) as previously described [87]. Cinnamon bark methanol extracts (2 μL) were injected on an HSS T3 column (100 × 1.0 mm, particle size 1.8 μm; Waters, Milford, MA, USA) installed on an ACQUITY UPLC system (Waters, Milford, MA, USA) equipped with a 6540 Agilent Ultra-High-Definition (UHD) Accurate-Mass Q-TOF-LC-MS (Palo Alto, CA, USA) coupled to an ESI interface, operated in positive or negative ion mode under the exact conditions of our previous work [56]. Characterization of metabolites was performed using their UV–VIS spectra (220–600 nm), exact masses, in addition to MS^2^ in both ionization modes, RT data, and reference literature and searching the phytochemical dictionary of natural products [88].

### 3.4. GC-MS Analysis of Silylated Primary Metabolites and Identification

Dried finely pulverized cinnamon specimens (100 mg) were extracted by adding 5 mL 100% MeOH, sonicated for 30 min with frequent shaking, then centrifuged at 12,000× *g* for 10 min to remove debris. Next, 100 µL of the methanol extract was transferred into screw-cap vials and evaporated under nitrogen gas until complete dryness. Then, 150 µL of MSTFA (*N*-methyl-*N*-(trimethylsilyl)-trifluoroacetamide), previously diluted 1:1 (*v/v*) with anhydrous pyridine, was added and incubated for 45 min at 60 °C for derivatization. Silylated products were separated by an Rtx-5MS column (30 m length, 0.25 mm i.d., and 0.25 µm film) [89]. For evaluation of biological replicates, under the same conditions, three separate samples were analyzed for each cinnamon specimen. Non-volatile silylated components were identified by comparing their Kovats indices (KI) relative to the C6-C20 *n*-alkane series, as well as matching the mass spectra obtained with the NIST and WILEY libraries and with standards when available. Before mass spectral matching, peaks were first deconvoluted through AMDIS software (www.amdis.net, accessed on 16 October 2020), and their abundance data were extracted using the MET-IDEA tool [90].

### 3.5. Multivariate Data (MVA) and Statistical Analyses

Each cinnamon group’s data were represented as the mean ± standard deviation (SD) of three replicates. One-way analysis of variance (ANOVA) was employed through IBM SPSS Statistics, Version 28.0. (Armonk, NY, USA: IBM Corp) with a *p*-value less than 0.05 to indicate significance between groups. The data table of MS abundances generated from either UPLC-MS or GC-MS was subjected to modeling, i.e., PCA (principal component analysis), HCA (hierarchical clustering analysis), and OPLS-DA (partial least-squares discriminant analysis) using SIMCA-P version 13.0 software package (Umetrics, Umeå, Sweden). Subsequently, markers were determined by analyzing the S-plot, which revealed covariance (p) and correlation (pcor). All variables were Pareto scaled and mean-centered. Validation of models was evaluated by computing the diagnostic indices, i.e., Q2 and R2 values, and permutation testing of iterations.

## 4. Conclusions

This study provides the most holistic map of cinnamon spice primary and secondary metabolites composition using a multiplex approach of UPLC-MS and GC-MS techniques analyzed using chemometric tools. Such metabolite profiling justifies the premium value of *C. verum* as a flavoring agent and in functional foods. UPLC-MS analysis allowed the identification of 74 metabolites, of which a new proanthocyanidin suggested to encompass catechin, chrysin, catechin, and hexose was detected for the first time, trihydroxylated fatty acid (trihydroxyoctadecaenoic acid) and three dicarboxylic fatty acids (hexadecanedioic acid, octadecenedioic acid, and hexadecanedioic acid methyl ester) were detected for the first time in cinnamon, albeit, though other spectroscopic analysis, i.e., NMR still required for complete elucidation of these metabolites. In addition, a number of newly identified flavonoid glycosides included naringenin di-*O*-hexoside, isorhamnetin-*O*-pentosyldeoxyhexoside, and luteolin-*O*-hexosyl-*C*-hexoside. It revealed the richness of Chinese cinnamon in coumarin, while *C. verum* and *C. tamala* were rich sources of cinnamates. Norboldine, an aporphine alkaloid of potential inhibitory activity against type I HIV, was detected at high levels in *C. iners* species, warranting further assays of its extract against different viruses. Despite the great proximity between *C. verum* of both origins, UPLC-MS allowed the detection of a number of compounds that accounted for differences between both origins, including dihydrocoumaroyl-*O*-hexoside and lignans. The palatability and agreeable taste of cinnamon spice pose it as an ingredient in nutraceuticals. According to the UPLC-MS profile, *C. iners* was the closest species to official *C. verum* concurrent with a low level of coumarin with a relatively high level of cinnamaldehyde, suggesting the former as a potential substitute for true cinnamon regarding minimal health hazards.

Primary metabolites analysis by GC-MS revealed true cinnamon richness in fatty acids and acyl esters, though with qualitative variation among different origins. Our findings also revealed that sugars were the most discriminatory metabolites among *Cinnamomum* species, with true cinnamon encompassing the highest levels compared to other specimens. Whereas *C. iners* showed the healthiest low-calorie sugar profile with lower sugars and high sugar alcohol levels at 29%, *viz*., *meso*-erythritol (16%) and arabitol (6%) and thus posing it as a sugar source for diabetics.

MVA of GC-MS and UPLC-MS detected in negative ion mode data revealed that *C. tamala* was the most chemically distinctive species attributed to the elevated dihydrocinnacasside pentoside, protocatechuic acid, and glycerol. In contrast, positive ion UPLC-MS mode revealed that *C. iners* was the most distant species, as it is rich in catechins and alkaloids, i.e., norboldine and norisocorydine. Among GC-MS and UPLC-MS employed analytical platforms, UPLC-MS in negative ion mode provided the most rational classification, with close segregation of CV and CVM specimens, and not observed in other PCA models. Novel markers revealed from this study to identify adulteration of true cinnamon (CV) with Chinese cinnamon (CC) included dihydrocoumaroyl-*O*-hexoside and dihydrocoumaroyl-*O*-pentosylhexoside in addition to the well-recognized coumarin. On the other hand, cinnamates represented by methyl cinnamate, (*E*)-cinnamaldehyde, and cinnamoyl alcohol were enriched in true cinnamon. Such chemical marker should aid in the detection of adulteration in true cinnamon, especially when present in extract lacking the typical morphological features to distinguish it from its allied drugs, i.e., Chinese type.

Although the selected *Cinnamomum* species do not represent all accessions of cinnamon worldwide, our approach is certainly feasible for analyzing other *Cinnamomum* species from such further sources to exploit factors that might impact the metabolic makeup, i.e., storage, seasonal variation and growth stage. Combining our variable metabolite profile data with gene expression can further assist in exploring involved genes, evaluating biosynthetic pathways, and ultimately enhancing breeding. The isolation and complete identification of the discriminative chemo-markers along with the newly highlighted metabolites should follow on as future work.

## Figures and Tables

**Table 1:** Metabolites identified in 70% methanol extract of *Cinnamomum* species by UPLC-ESI-MS (peak numbers are preceded by L in text) in both negative and positive ionization modes.

No.	Rt	Compound Name	Chemical Class	UV	[M − H]^−^/ [M + H]^+^	Molecular Formula	Error	MS/MS Fragments	Reference	CC	CI	CT	CV	CVM
L1	0.41	Hexose	Sugar	244	179.0561	C_6_H_11_O_6_^−^	3.1	161.0422 135.0323		+	+	+	+	+
L2	0.49	Quinic acid	Phenolic acid	222 264	191.0551	C_7_H_11_O_6_^−^	5.5	173.0418 129.0186	[28]	+	+	+	+	+
L3	0.72	Methoxycitric acid	Organic acid		221.0302 223.0145	C_7_H_9_O_8_^−^ C_7_H_11_O_8_^+^	0.4 0.6	189.0034 145.0131 127.0038		+	−	−	−	−
L4	1.55	Protocatechuic acid hexoside	Phenolic acid	223 280	315.0710	C_13_H_15_O_9_^−^	3.7	153.0185 109.0292	[29]	−	−	+	−	−
L5	1.62	Gentisic acid	Phenolic acid	280	153.0188 155.0206	C_7_H_5_O_4_^−^ C_7_H_7_O_4_^+^	1.4	109.0291		+	+	+	+	−
L6	1.82	Protocatechuic acid	Phenolic acid	280	153.0189	C_7_H_5_O_4_^−^	0.4	-	[30]	+	+	+	+	+
L7	2.41	Protocatechualdehyde	Phenolic aldehyde		137.0233	C_7_H_5_O_3_^−^	0.9	-	[29]	+	+	+	+	+
L8	2.62	Cinnamolaurine	Alkaloid	270 310	298.1433	C_18_H_19_NO_3_^+^	1.7	-	[31]	−	+	−	−	−
L9	2.88	(Epi)catechin tetramer (EC-EC-EC-A-EC)	Proanthocyanidin	234 275	1151.2454 1153.2626	C_60_H_47_O_24_^−^ C_60_H_49_O_24_^+^	0.8	863.1824 575.1204	[32]	+	+	−	−	−
L10	3.20	(Epi)catechin tetramer (EC-EC-A-EC-EC)	Proanthocyanidin	234 275	1151.25	C_60_H_47_O_24_^−^	−3.2	863.1870 573.1048		+	−	−	−	−
L11	3.27	(Epi)catechin trimer A type (EC-A-EC-EC)	Proanthocyanidin	234 280	863.1885	C_45_H_35_O_18_^−^	−6.5	711.1297 573.1144 289.0711		+	+	+	−	−
L12	3.40	(Epi)catechin trimer A type (EC-A-EC-EC)	Proanthocyanidin	280	863.1853 865.1958	C_45_H_35_O_18_^−^ C_45_H_37_O_18_^+^	0.6	577.1333 427.1819		+	+	+	−	−
L13	3.43	Dimethoxyphenol-*O*-pentosyl hexoside	Phenol	214	447.1501	C_19_H_27_O_12_^−^	1.6	269.1029 161.0448	[33]	+	+	+	+	+
L14	3.55	Norboldine	Alkaloids	220 280 310	312.1241 314.1382	C_18_H_18_NO_4_^−^ C_18_H_20_NO_4_^+^	2.7	297.0998	[34] [35]	+	+	+	+	+
L15	3.62	Phenylethyl*-O*-pentosyl hexoside (Icariside DC)	Hydroxycinnamates	214	415.1237	C_19_H_27_O_10_^−^	0.4	269.1034	[36]	+	−	+	+	+
L16	3.64	Dihydrocinnacasside pentoside	Hydroxycinnamates	214	459.1481	C_20_H_27_O_12_^−^	6	165.0552		−	+	+	+	+
L17	3.66	Dihydro coumaroyl-*O*-pentosylhexoside	Hydroxycinnamates	214	459.1506	C_20_H_27_O_12_^−^	0.5	415.1240 327.1078 165.0545		+	−	−	+	+
L18	3.67	Dihydro coumaroyl-*O*-hexoside (Dihydomelilotoside)	Hydroxycinnamates	214	327.1085 329.1034	C_15_H_19_O_8_^−^ C_15_H_21_O_8_^+^	0.7	281.1395 165.0544	[36]	+	−	+	+	−
L19	3.78	Dihydrocinnacasside (Hydroxyphenylpropanoy-*O*-hexoside)	Hydroxycinnamates	214	327.1063	C_15_H_19_O_8_^−^	6.8	165.055 121.0675		+	−	+	+	+
L20	3.84	Cinnamyl-*O*-hexoside	Hydroxycinnamates	280	295.1155	C_15_H_19_O_6_^−^	10.7	251.1266	[37]	−	−	+	+	−
L21	3.84	Corydine	Alkaloids	270 310	342.1678	C_20_H_24_NO_4_^+^	6.3	297.1106 265.0842	[38] [39]	+	+	+	+	+
L22	3.97	Reticuline	Alkaloids	280	330.1680	C_19_H_24_NO_4_^+^	5.9	192.1014		+	+	+	+	+
L23	4.16	Norisocorydine/ Boldine	Alkaloids	270 310	328.1528	C_19_H_22_NO_4_^+^	0.9	−		+	+	+	+	+
L24	4.50	Cinnamyl-*O*-pentosylhexoside	Hydroxycinnamates	251 280	427.1673	C_20_H_27_O_10_^−^	−6.3	293.0854 233.0659 149.0447	[40] [41]	+	−	−	−	−
L25	4.59	(Epi) catechin trimer with double A linkage	Proanthocyanidins	280	861.699 863.1811	C_45_H_33_O_18_^−^ C_45_H_35_O_18_^+^	−2.7	595.1699 575.118 473.1653	[42]	+	−	+	−	−
L26	4.60	Dihydrocoumaric acid	Hydroxycinnamates	310	165.0555	C_9_H_9_O_3_^−^	12.9	121.0657	[43]	+	+	+	+	−
L27	4.63	Dihydrocinnamyl-*O*-pentosyl hexoside	Hydroxycinnamates	251 280	429.1726	C_20_H_31_O_12_^−^	1	297.1323 149.0440	[41]	−	+	−	−	−
L28	4.68	Naringenin di-*O*-hexoside	Flavonoids		595.1699 597.1254	C_27_H_31_O_15_^−^ C_27_H_33_O_15_^+^	−20.4	433.1139 271.0611	[44]	−	+	+	−	−
L29	4.85	Isorhamnetin-*O*-pentosyldeoxyhexoside	Flavonoids	256 354	593.1848	C_28_H_33_O_14_^−^	4.7	447.1285 315.0701		+	−	+	−	−
L30	4.93	Luteolin-*O*-hexoside-*C*-hexoside	Flavonoids	260 348	609.1998	C_27_H_29_O_16_^−^	−5.2	447.0924 327.1076		+	−	+	−	−
L31	5.12	Dipropylmalonic acid	Organic acid		187.0973 189.0721	C_9_H_15_O_4_^−^ C_9_H_17_O_4_^+^	1.3	169.0866 143.1078		+	+	+	+	+
L32	5.15	Trimethoxy phenol	Phenol		183.0655 185.0712	C_9_H_11_O_4_^−^ C_9_H_13_O_4_^+^	4.2	155.0708 139.0758		+	+	+	+	+
L33	5.32	Coumarin	Hydroxycinnamates	273 312	147.0446	C_9_H_6_O_2_^+^	−3.8		[45]	+	+	+	+	+
L34	5.46	Dihydroxy-tetramethoxy-epoxylignanolone	Lignans	233 303	433.1479	C_22_H_25_O_9_^−^	5.6	418.1258 373.1270 285.0428	[46]	+	+	+	+	+
L35	5.61	Oxododecanedioic acid	Fatty acids		243.1228	C_12_H_19_O_5_^−^	4	225.1129 181.1215	[47]	−	+	+	+	+
L36	5.78	Acetyl methoxy coumarin	Hydroxycinnamates		217.0501	C_12_H_9_O_4_^−^	2.5	185.0814 173.0600	[48]	+	+	+	+	+
L37	6.09	Unknown	Catechins	234 280	995.2414	C_51_H_47_O_21_^−^	−29.1	705.1589 543.1298 289.0178		−	−	+	−	−
L38	6.13	Cinnamic acid (*E*-cinnamic acid)	Hydroxycinnamates	270	147.0435 149.0723	C_9_H_7_O_2_^−^ C_9_H_9_O_2_^+^	4.5	119.0503	[49]	+	+	+	+	+
L39	6.50	(*E*)-Cinnamaldehyde	Hydroxycinnamates		133.0652	C_9_H_9_O^+^	−3.3	-	[22]	+	+	+	+	+
L40	7.11	Unknown	nitrogenous compound		242.1751 244.1574	C_13_H_24_NO_3_^−^ C_13_H_26_NO_3_^+^	4.5	225.1502		+	+	+	+	+
L41	7.15	Methylcinnamic acid	Hydroxycinnamates		163.074	C_10_H_11_O_2_^+^		-	[50]	+	+	−	+	+
L42	7.33	Methoxy cinnamaldehyde	Hydroxycinnamates		163.076	C_10_H_11_O_2_^+^	0.3	-		+	+	+	+	+
L43	7.30	Cinnamyl alcohol	Hydroxycinnamates		135.081	C_9_H_11_O^+^	−3.8	-	[50]	+	+	+	+	+
L44	7.51	Hydroxyl, dimethoxyphenyl, hydroxy methoxyphenyl propanediol	Phenol		349.12	C_18_H_22_O_7_^−^	4.7	331.1177 293.1388 225.0767	[51]	−	+	−	+	+
L45	7.52	Epicatechin trimethyl ether	Catechins		333.1323	C_18_H_21_O_6_^+^	2.8	-	[52]	+	+	+	+	+
L46	8.02	Cinnacassin L	Lignans	215 245	281.1168	C_17_H_18_O_3_^−^	5.6	207.1183 147.0448	[51]	−	+	−	+	−
L47	8.80	Physcion	Anthraquinon		283.0606	C_16_H_11_O_5_^−^	2	269.0381	[53]	−	+	−	−	+
L48	9.00	Methylenedioxy-dimethylepicatechin	Catechins	234 280	331.117	C_18_H_19_O_6_^+^	1.8	-		+	+	+	+	+
L49	9.43	Hydroperoxy-linolenate	Fatty acid		309.2047 311.2137	C_18_H_29_O_4_^−^ C_18_H_31_O_4_^+^	7.9	291.1967 265.2163		−	+	+	+	−
L50	9.83	Dihydrocapsiate (vanillyl-8-methylnonanate)	Methoxyphenols		307.1919	C_18_H_27_O_4_^−^	−1.5	265.1800 223.1331 209.1180	[54]	+	+	+	+	−
L51	9.92	(Epi)gallocatechin-*O*-caffeate	Proanthocyanidins	234 280	467.0982	C_24_H_19_O_10_^−^	0.3	313.2369 161.0243		−	−	+	−	−
L52	9.99	(Epi)gallocatechin-(epi)catechin	Proanthocyanidins	278	593.2696	C_30_H_41_O_12_^−^	−15.6	467.0971 313.2373 305.1775	[55]	−	−	+	−	−
L53	10.52	Hexadecanedioic acid	Fatty acid		285.2060 287.2163	C_16_H_30_O_4_^−^ C_16_H_32_O_4_^+^	4.4	267.1947 223.2042	[56]	−	+	−	+	+
L54	11.12	Octadecenedioic acid	Fatty acid		311.2205 313.2147	C_18_H_31_O_4_^−^ C_18_H_33_O_4_^+^	5.1	293 249.2234		+	+	+	+	+
L55	11.36	Hydroxylinoleic acid	Fatty acid	221	295.2267	C_18_H_31_O_3_^−^	4.1	277.2161 195.1379		−	−	+	+	+
L56	12.30	Emodin	Anthraquinone		269.2091	C_15_H_10_O_5_^−^		225.2199	[53]	+	+	+	+	+
L57	12.85	Hexadecanedioic acid, monomethyl ester (Methoxy-oxohexadecanoic acid)	Fatty acid	221	299.2060	C_17_H_31_O_4_^−^	1.4	255.2320		+	+	+	−	+
L58	12.96	Hydroperoxyoctadecenoic acid	Fatty acid		313.2373	C_18_H_33_O_4_^−^	4.2	269.2297	[54]	+	+	+	+	+
L59	13.97	Ceriporic acid	Dicarboxylic acid		351.2534 353.2431	C_21_H_35_O_4_^−^ C_21_H_37_O_4_^+^	1.9	-		+	+	−	+	+
L60	14.02	Trihydroxyoctadecadienoic acid	Fatty acid	221	327.2177 329.1743	C_18_H_31_O_5_^−^ C_18_H_33_O_5_^+^	3.2	283.2266	[54]	−	+	−	+	+
L61	14.25	Hydroxyoctadecenoic acid	Fatty acid		297.2405	C_18_H_33_O_3_^−^	10.1	253.2167 235.2058		+	+	+	+	+
L62	14.28	Cinnakotolactone	Lactone		309.2426 311.2541	C_19_H_33_O_3_^−^ C_19_H_35_O_3_^+^	3.7	-	[57]	+	−	+	+	+
L63	14.39	Cinnamyl cinnamate-*O*-pentoside	Hydroxycinnamates		395.2781	C_23_H_39_O_5_^−^	2.2	263.2389		−	+	−	−	−
L64	14.54	Linoleic acid	Fatty acid	221	279.232 281.2231	C_18_H_31_O_2_^−^ C_18_H_33_O_2_^+^	2	235.2051	[58]	+	+	+	+	+
L65	14.99	Isolinderanolide	Butanolides		307.2272 309.2103	C_19_H_31_O_3_^−^ C_19_H_33_O_3_^+^	5.1	-	[57]	+	+	+	+	+
L66	15.25	Palmitic acid	Fatty acid	221	255.2328 257.2167	C_16_H_31_O_2_^−^ C_16_H_33_O_2_^+^	0.7	-	[58]	+	+	+	+	+
L67	15.40	Cinncassiol B	Diterpene		399.1961	C_20_H_31_O_8_^−^	15.4	-	[59]	+	+	+	+	+
L68	15.53	Cinncassiol A	Diterpene		381.1724	C_20_H_29_O_7_^−^	51	337.1820		+	+	+	+	+
L69	15.54	Oleic acid	Fatty acid	224	281.2483 283.2641	C_18_H_33_O_2_^−^ C_18_H_35_O_2_^+^	0.9	-	[58]	+	+	+	+	+
L70	15.75	Benzenedicarboxylic acid, bis(2-methylpropyl) ester	Fatty ester		279.1587	C_16_H_23_O_4_^+^	0.4	-	[60]	+	+	+	+	+
L71	15.81	Methyl palmitate	Fatty acid	221	271.2626	C_17_H_35_O_2_^+^	0.6	-		+	+	+	+	+
L72	15.84	Olealdehyde	Fatty aldehydes		267.2677	C_18_H_35_O^+^	2.1	-		+	+	+	+	+
L73	17.03	Benzenedicarboxylicacid, diisooctylester	Fatty ester		391.2795	C_24_H_39_O_4_^+^	0.4	-	[60]	+	+	+	+	+
L74	18.11	Hydroxy-dimethoxy epoxy-neolignene diolethyl ether	Lignans	205 303	385.1657	C_22_H_26_O_6_^−^	18.6	-	[46]	−	+	+	+	−

CC: *Cinnamomum cassia* from Malaysia, CI: *C. iners* from Malaysia, CT: *C. tamala* from Pakistan, CV: *C. verum* from Pakistan, CVM*: C. verum* from Malaysia.

**Table 2:** Relative percentage of non-volatile metabolites detected in cinnamon barks using HS-SPME-GC-MS (peak numbers are preceded by G in text) measurements (*n* = 3) represented as average ± standard errors. Different letters indicate significant differences between cinnamon accessions according to the least significant difference analysis (*p* < 0.05; Tukey’s test). CC: *Cinnamomum cassia* from Malaysia, CI: *C. iners* from Malaysia, CT: *C. tamala* from Pakistan, CV: *C. verum* from Pakistan, CVM*: C. verum* from Malaysia. ^(**a**)–(**e**)^ significantly different form the corresponding group. * Compounds confirmed by standards comparison.

No.	Rt (min)	RI	Identification	CC ^(a)^	CI ^(b)^	CT ^(c)^	CV ^(d)^	CVM ^(e)^
**Amino acids**								
G1	12.218	1403	L-Aspartic acid, 2TMS	2.27 ± 0.36	2.03 ± 0.36	1.36 ± 0.15	2.27 ± 0.21	1.48 ± 0.08
G2	12.652	1436	*β*-Alanine, 3TMS *	0.20 ± 0.04	0.18 ± 0.02	0.11 ± 0.00	0.23 ± 0.02	0.13 ± 0.01
Total Amino acids				2.47	2.21	1.47	2.49	1.60
**Esters**								
G3	24.632	2602	1-Monopalmitin, 2TMS	6.06 ± 3.88	10.43 ± 0.38	5.03 ± 1.06	9.03 ± 2.13	3.72 ± 1.79
G4	26.106	2789	Glycerol monostearate, 2TMS	15.13 ± 5.57	21.73 ± 0.52 ^e^	12.01 ± 1.21 ^d^	23.96 ± 2.41 ^c^	11.46 ± 2.50 ^b^
G5	26.322	2811	Sebacic acid di(2-ethylhexyl) ester	0.59 ± 0.01	0.61 ± 0.02	0.33 ± 0.01	0.63 ± 0.03	0.36 ± 0.06
Total esters				21.79	32.77	17.37	33.61	15.54
**Ethers**								
G6	9.918	1253	Diethylene glycol, 2TMS	0.25 ± 0.02	0.25 ± 0.01	0.15 ± 0.01	0.27 ± 0.01	0.15 ± 0.02
Total ethers				0.25	0.25	0.15	0.27	0.15
**Fatty acids**								
G7	17.733	1850	Myristic acid, TMS *	0.33 ± 0.01	0.59 ± 0.03	0.28 ± 0.01	0.47 ± 0.03	0.31 ± 0.03
G8	18.784	1949	Pentadecanoic acid, TMS	0.09 ± 0.01	0.33 ± 0.02	0.07 ± 0.00	0.20 ± 0.03	0.07 ± 0.02
G9	19.779	2047	Palmitic Acid, TMS *	2.33 ± 0.15	2.65 ± 0.06	2.48 ± 0.16	4.10 ± 0.36	1.77 ± 0.41
G10	21.406	2216	Linoleic acid, TMS *	0.03 ± 0.00	0.09 ± 0.01	0.04 ± 0.01	0.22 ± 0.09	0.09 ± 0.05
G11	21.44	2220	Oleic Acid, TMS *	0.69 ± 0.03	0.84 ± 0.08	0.92 ± 0.11	2.09 ± 0.74	0.67 ± 0.28
G12	21.495	2226	Elaidic acid TMS	0.29 ± 0.01	0.24 ± 0.03	0.19 ± 0.04	0.39 ± 0.11	0.22 ± 0.04
G13	21.655	2244	Stearic acid, TMS	3.35 ± 0.10	3.60 ± 0.04	2.11 ± 0.14	3.94 ± 0.20	2.08 ± 0.35
G14	23.386	2444	Arachidic acid, TMS	0.22 ± 0.01	0.15 ± 0.01	0.14 ± 0.04	0.22 ± 0.04	0.11 ± 0.04
G15	24.983	2646	Behenic acid, TMS	0.55 ± 0.07	0.28 ± 0.05	0.29 ± 0.12	0.41 ± 0.08	0.22 ± 0.12
G16	26.466	2824	Lignoceric acid, TMS	0.25 ± 0.03	0.18 ± 0.01	0.17 ± 0.04	0.25 ± 0.02	0.13 ± 0.05
Total fatty acids				8.14	8.94	6.70	12.30	5.68
**Organic acids**								
G17	7.06	1076	Glycolic acid, 2TMS	0.19 ± 0.03	0.24 ± 0.01	0.25 ± 0.04	0.25 ± 0.01	0.12 ± 0.02
G18	7.549	1113	Oxalic acid, 2TMS	1.63 ± 0.45	1.57 ± 0.32	0.48 ± 0.07	1.33 ± 0.36	1.10 ± 0.39
G19	8.265	1153	3-hydroxypropionic acid, 2TMS	0.18 ± 0.04	0.11 ± 0.01	0.42 ± 0.02	0.16 ± 0.01	0.14 ± 0.02
G20	9.766	1243	4-hydroxybutyric acid, 2TMS	1.01 ± 0.25	0.82 ± 0.10	0.52 ± 0.04	0.98 ± 0.10	0.10 ± 0.01
G21	10.983	1322	Succinic acid, 2TMS	0.60 ± 0.04	2.08 ± 0.00	0.82 ± 0.04	0.47 ± 0.02	0.36 ± 0.04
G22	13.529	1502	Malic acid, 3TMS *	0.64 ± 0.01	1.41 ± 0.21	1.52 ± 0.13	1.50 ± 0.20	0.49 ± 0.04
G23	14.269	1557	(*E*)-Cinnamic acid, TMS *	0.41 ± 0.05	0.21 ± 0.02	1.02 ± 0.10	0.10 ± 0.01	0.70 ± 0.08
G24	17.418	1821	Shikimic acid, 4TMS	0.44 ± 0.05	0.37 ± 0.02	0.90 ± 0.01	0.25 ± 0.02	0.26 ± 0.04
G25	18.099	1885	Quinic acid, 5TMS	1.10 ± 0.10	1.05 ± 0.03	2.03 ± 0.15	0.45 ± 0.08	0.44 ± 0.06
Total organic acids				6.20	7.86	7.96	5.49	3.70
**Phenolics**								
G26	17.543	1832	Protocatechuic acid, 3TMS	0.63 ± 0.13 ^c,d^	0.26 ± 0.01^c,d^	4.52 ± 0.27 ^a,b,d,e^	0.19 ± 0.17 ^a,b,c,e^	0.36 ± 0.02 ^c,d^
G27	26.849	2859	Catechin, 5TMS	0.35 ± 0.09	0.35 ± 0.06	0.10 ± 0.03	1.40 ± 0.03	0.16 ± 0.03
Total phenolics				0.98	0.61	4.62	1.59	0.52
**Sugars**								
G28	15.63	1667	Arabinose, 4TMS	0.62 ± 0.09	0.19 ± 0.04	0.56 ± 0.01	0.26 ± 0.03	0.13 ± 0.02
G29	17.476	1826	Psicofuranose, 5TMS	1.96 ± 0.23 ^b,c,d,e^	0.11 ± 0.03 ^a,c,e^	1.06 ± 0.15 ^a,b,e^	0.92 ± 0.22 ^a,e^	2.94 ± 0.44 ^a,b,c,d^
G30	17.557	1834	Psicofuranose, 5TMS isomer	5.46 ± 0.60 ^b,d,e^	0.58 ± 0.04 ^a,c,d,e^	6.86 ± 0.11 ^b,d^	3.01 ± 0.46 ^a,b,c,e^	7.54 ± 1.11 ^a,b,d^
G31	17.662	1844	Fructopyranose, 5TMS *	8.45 ± 1.23 ^b,d,e^	0.45 ± 0.01 ^a,c,d,e^	6.30 ± 0.40 ^b,e^	4.21 ± 0.44 ^a,b,e^	12.93 ± 0.58 ^a,b,c,d^
G32	18.45	1918	Glucopyranose, 5TMS *	8.10 ±1.01 ^b,c,d,e^	0.27 ± 0.01 ^a,c,d,e^	5.98 ±0.47 ^a,b,d,e^	3.73 ± 0.53 ^a,b,c,e^	10.97 ± 0.68 ^a,b,c,d^
G33	19.347	2002	Glucopyranose, 5TMS isomer	10.01 ± 1.46 ^b,c,d,e^	0.37 ± 0.04 ^a,c,d,e^	7.43 ± 0.35 ^a,b,d,e^	4.63 ± 0.49 ^a,b,c,e^	12.51 ± 0.36 ^a,b,c,d^
G34	25.318	2689	Sucrose, 8TMS *	2.35 ± 0.28	0.25 ± 0.04	0.83 ± 0.19	0.77 ± 0.36	0.25 ± 0.07
Total sugars				36.95	2.23	29.03	17.52	47.27
**Sugar acids**								
G35	11.338	1343	Glyceric acid, 3TMS	0.25 ± 0.05	0.22 ± 0.01	0.16 ± 0.01	0.37 ± 0.02	0.15 ± 0.02
G36	14.529	1577	Erythronic acid, 4TMS	0.03 ± 0.00	0.05 ± 0.01	0.06 ± 0.00	0.08 ± 0.01	0.01 ± 0.00
G37	18.381	1909	Galactonic acid, γ-lactone, 4TMS	1.74 ± 0.33 ^b,d,e^	0.13 ± 0.04 ^a,c,e^	2.02 ± 0.12 ^b,d^	0.74 ± 0.15 ^a,c,e^	2.78 ± 0.42 ^a,b,d^
G38	18.493	1923	Galactonic acid, γ-lactone, 4TMS isomer	0.11 ± 0.02	0.28 ± 0.09	0.71 ± 0.01	0.11 ± 0.02	9.03 ± 1.06
G39	18.732	1944	Talonic acid, γ-lactone, 4TMS	0.03 ± 0.01	0.01 ± 0.00	0.01 ± 0.00	0.03 ± 0.00	0.01 ± 0.00
G40	19.627	2031	Gluconic acid, 6TMS	0.32 ± 0.02	1.87 ± 0.07	1.56 ± 0.27	0.61 ± 0.09	0.10 ± 0.02
Total sugar acids				2.47	2.56	4.52	1.94	12.09
**Sugar alcohols**								
G41	10.434	1286	Glycerol, 3TMS *	3.60 ± 0.64 ^c^	2.56 ± 0.14 ^c,d^	11.29 ± 0.98 ^a,b,d,e^	4.63 ±0.38 ^b,c,e^	2.36 ±0.37 ^c,d^
G42	13.721	1516	L-Threitol, 4TMS	0.04 ± 0.01	0.19 ± 0.01	0.20 ± 0.00	0.05 ± 0.00	0.04 ± 0.01
G43	13.819	1524	meso-Erythritol, 4TMS	1.43 ± 0.26 ^b,c,d^	15.96 ± 0.28 ^a,c,d,e^	3.84 ±0.12 ^a,b,d,e^	2.61 ± 0.06 ^a,b,c,e^	1.04 ± 0.14 ^b,c,d^
G44	16.362	1729	Arabitol, 5TMS	0.06 ± 0.01	0.06 ± 0.01	0.14 ± 0.01	0.04 ± 0.00	0.03 ± 0.00
G45	16.502	1741	Arabitol, 5TMS isomer	0.54 ± 0.08	6.14 ± 0.24	2.13 ± 0.09	0.33 ± 0.02	0.13 ± 0.01
G46	18.944	1965	Mannitol, 6TMS	0.11 ± 0.02	0.17 ± 0.04	0.42 ± 0.03	0.99 ± 0.11	0.28 ± 0.03
G47	19.133	1982	Myo-Inositol, 6TMS *	0.08 ± 0.01	0.10 ± 0.00	0.12 ± 0.01	0.18 ± 0.02	0.13 ± 0.02
G48	20.5	2121	Myo-Inositol, 6TMS isomer	0.79 ± 0.11	3.49 ± 0.11	1.29 ± 0.04	0.72 ± 0.09	0.78 ± 0.11
Total sugar alcohols				6.67	28.68	19.42	9.57	4.80
**Unknowns**								
G49	10.903	1317	Unknown 1	3.08 ± 0.59	2.45 ± 0.23	1.66 ± 0.14	3.43 ± 0.12	1.94 ± 0.22
G50	11.596	1362	Unknown 2	10.23 ± 0.67	10.46 ± 0.13	5.82 ± 0.36	11.25 ± 0.33	6.34 ± 0.89
G51	19.881	2058	Unknown 3	0.77 ± 0.13	0.98 ± 0.01	1.27 ± 0.01	0.54 ± 0.08	0.37 ± 0.06
Total unknowns				14.07	13.89	8.76	15.22	8.65

## Data Availability

The data presented in this study are available in the Supplementary Material.

## Supplementary Materials

The following supporting information can be downloaded at https://www.mdpi.com/article/10.3390/molecules27092935/s1: Figure S1: MS^2^ Spectra of (epi) catechin tetramer (peak 9) [M − H]^−^ *m*/*z* 1151.2454, C_60_H_48_O_24_; Figure S2: MS^2^ Spectra of (epi) catechin tetramer (peak 10) [M − H]^−^ *m*/*z* 1151.25, C_60_H_48_O_24_; Figure S3: MS^2^ Spectra of A, B (epi) catechin trimer A type (peaks 11, 12) [M − H]^−^ *m*/*z* 863.1885, C_45_H_36_O_18_; Figure S4: MS^2^ Spectra of Catechin-chrysin-catechin-*O*-hexoside (peak 37) [M − H]^−^ *m*/*z* 995.2414, C_51_H_48_O_21_; Figure S5: MS^2^ Spectra of dihydrocinnamyl-*O*-pentosylhexoside (peak 27) [M − H]^−^ *m*/*z* 429.1762, C_20_H_32_O_12_; Figure S6: MS^2^ Spectra of luteolin-O/C-di-hexoside (peak 30) [M − H]^−^ *m*/*z* 609.1998, C_27_H_30_O_16_; Figure S7: MS^2^ Spectra of corydine (peak 21) [M − H]^−^ *m*/*z* 342.1678, C20H23NO4; Figure S8: MS^2^ Spectra of dicarboxylic fatty acids; Figure S9: UPLC-MS OPLS-DA, (a) score plot and (b) loading S-plots derived from modelling CT (*C. tamala* from Pakistan) against other samples in a separate group on negative ion mode. OPLS-DA (c) score plot and (d) loading S-plots derived from modeling CI (*C. iners* from Malaysia) against other samples in a separate group on positive ion mode; Figure S10: UPLC-MS OPLS-DA (a) score plot and (b) loading S-plots derived from modeling CC (*Cinnamomum cassia* from Malaysia) versus CV (*C. verum* from Pakistan) and CVM (*C. verum* from Malaysia) in a separate group on negative ion mode. OPLS-DA (c) score plot and (d) loading S-plots derived from modeling CC versus CV and CVM on positive ion mode. Each S-plot revealed the covariance p[1] against the correlation p(cor)[1] of the variables of the discriminating component; Figure S11: UPLC-MS OPLS-DA (a) score plot and (b) loading S-plots derived from modelling CV (*C. verum* from Pakistan) versus CVM (*C. verum* from Malaysia) on negative ion mode revealing the covariance p[1] against the correlation p(cor)[1] of the variables of the discriminating component; Figure S12: Representative SPME-GC-MS chromatograms of cinnamon primary metabolites, acquired from (a) CI (*C. iners* from Malaysia), (b) CT (*C. tamala* from Pakistan) and (c) CV (*C. verum* from Pakistan); Figure S13: GC-MS OPLS-DA (a) score plot and (b) loading S-plots derived from modelling CT (*C. tamala* from Pakistan) versus all other samples revealing the covariance p[1] against the correlation p(cor)[1] of the variables of the discriminating component; Figure S14: GC-MS OPLS-DA (a) score plot and (b) loading S-plots derived from modelling CC (*Cinnamomum cassia* from Malaysia) versus CV (*C. verum* from Pakistan) revealing the covariance p[1] against the correlation p(cor)[1] of the variables of the discriminating component; Table S1: Origin of the different species of cinnamon barks used in the analysis; Table S2: Relative quantification of the most discriminatory metabolites in the studied Cinnamomum species identified by UPLC-ESI-MS and multivariate analysis. Values are represented as average (*n* = 3) of normalized peak areas × 10^3^ to umbelliferon (internal standard) ± standard error. Different letters indicate significant differences between cinnamon accessions according to the least significant difference analysis (*p* < 0.05; Tukey’s test).

## Sample Availability

Samples of the compounds are not available from the authors.
