# Supplementary material for: Comparative Metabolite Fingerprinting of Four Different Cinnamon Species Analyzed via UPLC–MS and GC–MS and Chemometric Tools
Source: Molecules. 2022 May 4;27(9):2935. doi: 10.3390/molecules27092935 (PMC9104325; doi:10.3390/molecules27092935)
Supplement: Supplementary file 1 [file molecules-27-02935-s001.zip › molecules-1677357-supplementary.pdf]

Supplementary Materials

Table S1. Origin of the different species of cinnamon barks used in the analysis.

Name	Location	Sample codes
Cinnamomum cassia	Malaysia (Kuala Lumpur)	**CC**
Cinnamomum iners	Malaysia (Kuala Lumpur)	**CI**
Cinnamomum tamala	Pakistan (Karachi)	**CT**
Cinnamomum verum	Pakistan (Karachi)	**CV**
Cinnamomum verum	Malaysia (Kuala Lumpur)	**CVM**

Table S2. Relative quantification of the most discriminatory metabolites in the studied *Cinnamomum* species identified by UPLC-ESI-MS and multivariate analysis. Values are represented as average (n=3) of normalized peak areas $\times 10^3$ to umbelliferon (internal standard) ± standard error. Different letters indicate significant differences between cinnamon accessions according to least significant difference analysis ($p < 0.05$; Tukey's test).

No.	Rt(min)	Identification	CC [(a)]	CI [(b)]	CT [(c)]	CV [(d)]	CVM [(e)]
1	1.82	Protocatechuic acid	46.46 ± 0.79 [b,c,e]	302.42 ± 5.67 [a,c,d,e]	1110.37 ± 26.36 [a,b,d,e]	3.49 ± 3.84 [b, c, e]	180.3 ± 3.6 [a,b,c,d]
2	3.55	Norboldine	40.27±1.79 [b]	10078.11± 130.36 [a,c,d,e]	6.51±0.01 [b]	5.71±1.19 [b]	44.42±1.88 [b]
3	3.64	Dihydrocinnacasside-*O*-pentoside	00±00 [b,c,d,e]	29.54±0.42 [a,c,d,e]	366.53±5.15 [a,b,d,e]	13.71±0.55 [a,b,c,e]	26.37±1.18 [a,b,c,d]
4	3.67	Dihydro coumaroyl -*O*- hexoside	276.51±10 [b,c,d,e]	00±00 [a,c,d]	307.53±11.72 [a,b,d,e]	35.23±2.01 [a,b,c,e]	10.94±10.01 [a,c,d]
5	5.32	Coumarin	1258.21 ± 10.34 [d]	336.2 ± 13.21 [d,e]	2114.18 ± 30.25 [d]	947.48 ± 20.85 [a,b,c]	947.9 1± 10 [b]
6	6.13	Cinnamic acid	30.62±2.01 [b,c,d,e]	124.98± 23.18 [a,d,e]	129.85±15.85 [a,d,e]	1120.86±30.78 [a,b,c,e]	1917.9±7.29 [a,b,c,d]
7	6.5	*(E)*- Cinnamaldehyde	4.92 ± 1.29 [b,d,e]	1413.8 ± 38.29 [a,c,d,e]	52.3 ± 6.19 [b,d,e]	3424.28 ± 6.84 [a,b,c,e]	3831.26 ± 8.49 [a,b,c,d]
8	7.15	Methylcinnamic acid	00±00 [d,e]	226.9±5.36 [d,e]	00±00 [d,e]	13745.3±18.59 [a,b,c,e]	6390.19±15.96 [a,b,c,d]
9	9.00	Methylenedioxy-dimethylepicatechin	5.97±3.81 [b,c,d,e]	78.49±2.19 [a,c,e]	00±00 [a,b,d,e]	78.64±15.28 [a,c,e]	113.59±5.61 [a,b,c,d]
10	14.54	Linoleic acid	221.18 ± 7.09 [b,c,d,e]	2737.3 ± 51.69 [a,c,d,e]	473.19 ± 15.49 [a,b,d,e]	2065.69 ± 6.28 [a,b,c,e]	4371.19 ±4.59 [a,b,c,d]
11	15.25	Palmitic acid	156.09 ± 5.49 [b,c,d,e]	4340.74 ± 8.69 [a,c,d,e]	616.21 ± 10.89 [a,b,d,e]	4478.69 ± 3.19 [a,b,c,e]	1224.68 ± 20.49 [a,b,c,d]
12	15.54	Oleic acid	122.67 ± 0.04 [b,c,d,e]	1889.67 ± 8.36 [a,c,d]	655.4 ± 10.59 [a,b,d,e]	6391.06 ± 8.036 [a,b,c,e]	1993.61 ± 3.19 [a,c,d]

* CC: *Cinnamomum cassia* from Malaysia, CI: *C. iners* from Malaysia, CT: *C. tamala* from Pakistan, CV: *C. verum* from Pakistan, CVM*:* *C. verum* from Malaysia.

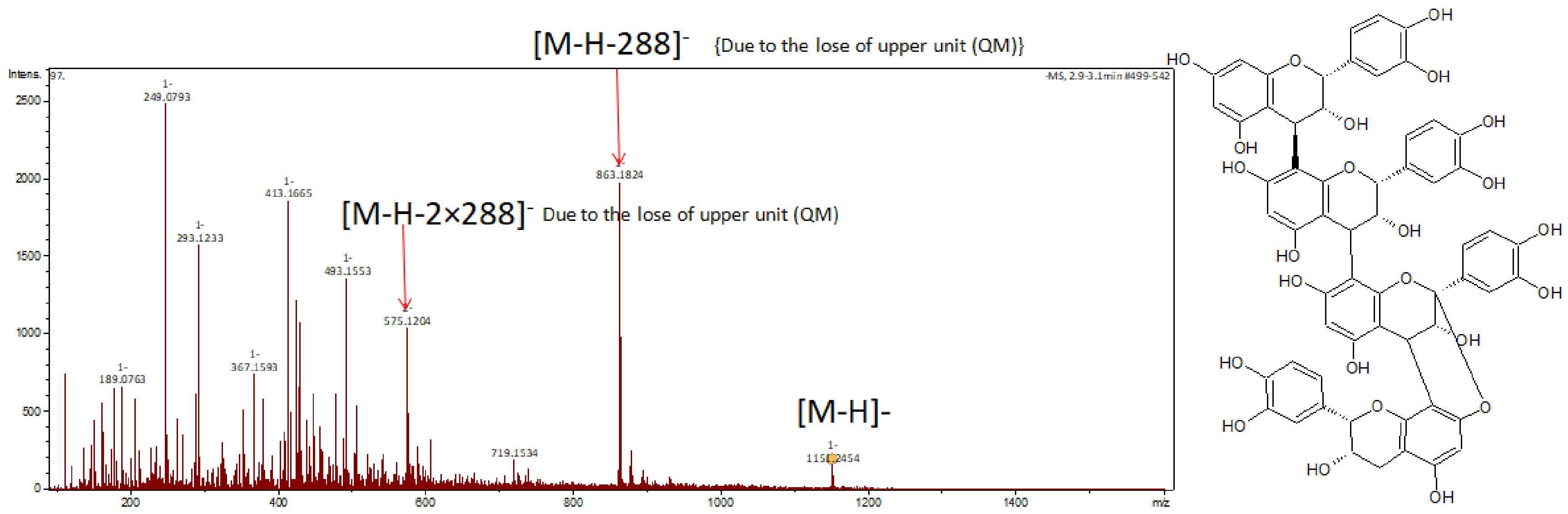

Figure S1. MS[2] Spectra of (epi) catechin tetramer (peak **9**) $[M-H]^-$ *m/z* 1151.2454, $C_{60}H_{48}O_{24}$

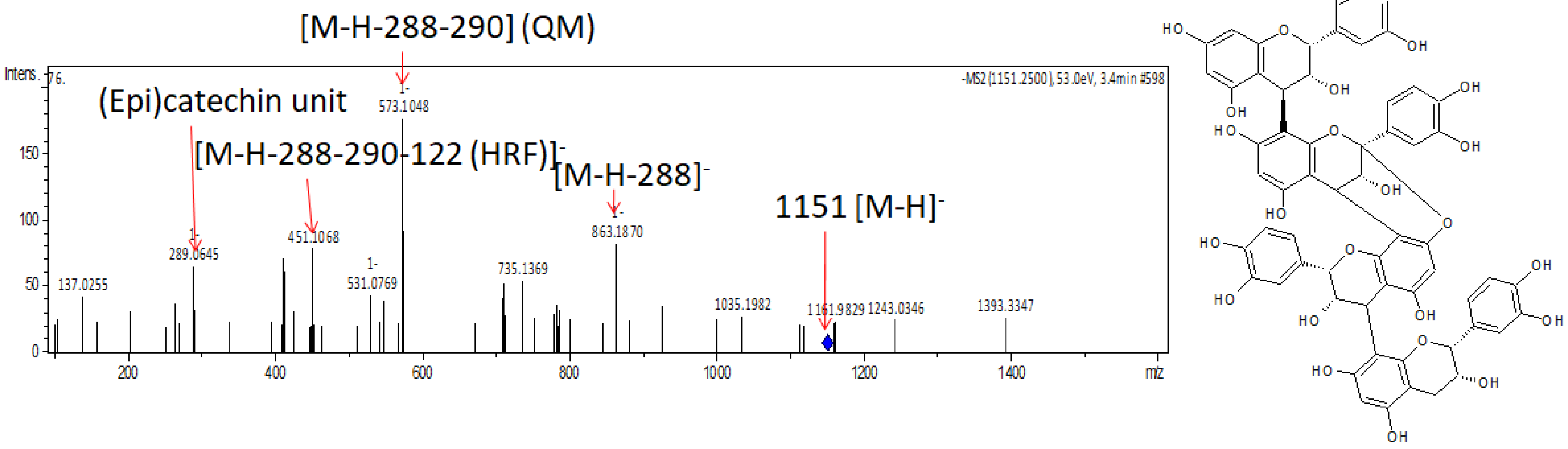

Figure S2. MS[2] Spectra of (epi) catechin tetramer (peak **10**) $[M-H]^-$ *m/z* 1151.25, $C_{60}H_{48}O_{24}$

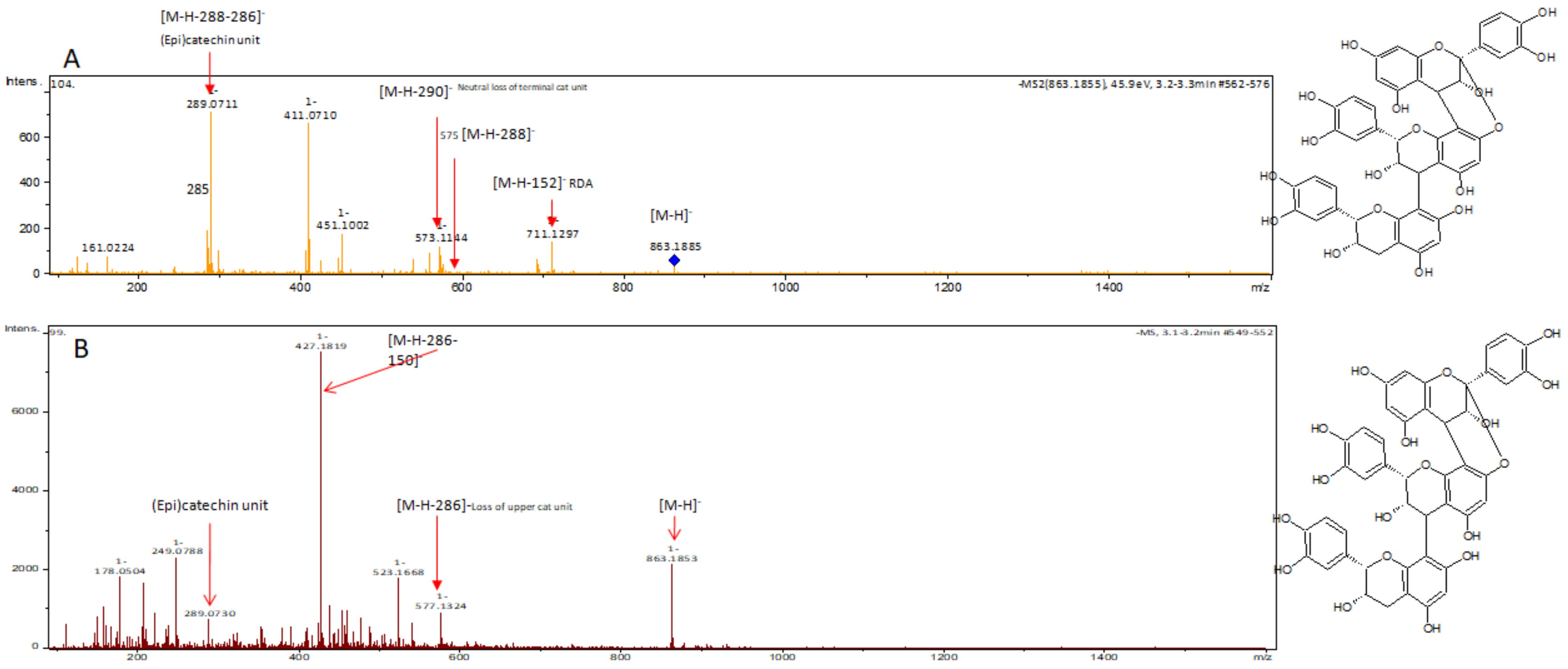

Figure S3. MS[2] Spectra of **A, B** (epi) catechin trimer A type (peaks **11**, **12**) $[M\text{-}H]^-$ *m/z* 863.1885, $C_{45}H_{36}O_{18}$

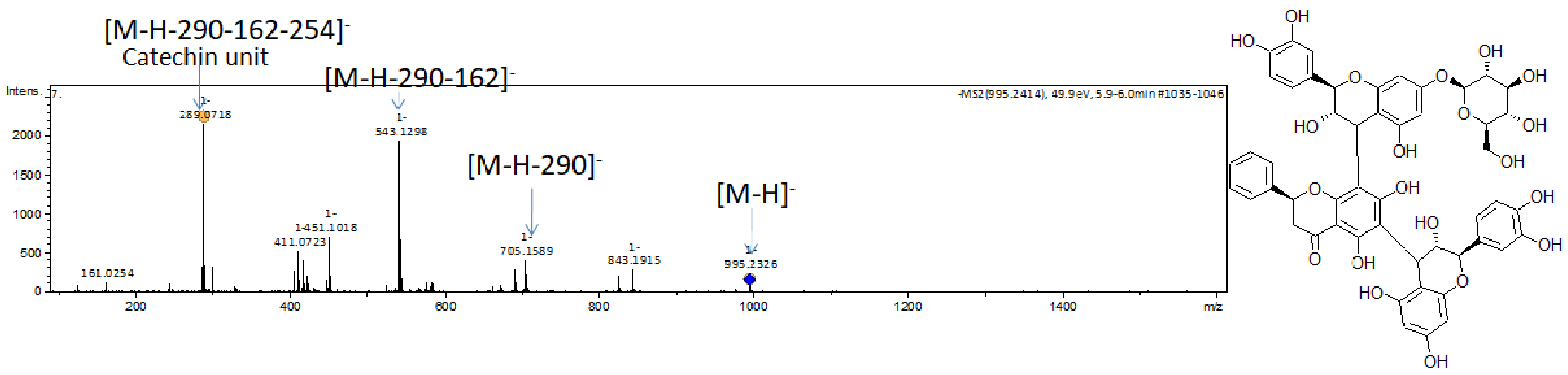

Figure S4. MS[2] Spectra of Catechin-chrysin-catechin-*O*-hexoside (peak **37**) $[M-H]^-$ *m/z* 995.2414, $C_{51}H_{48}O_{21}$

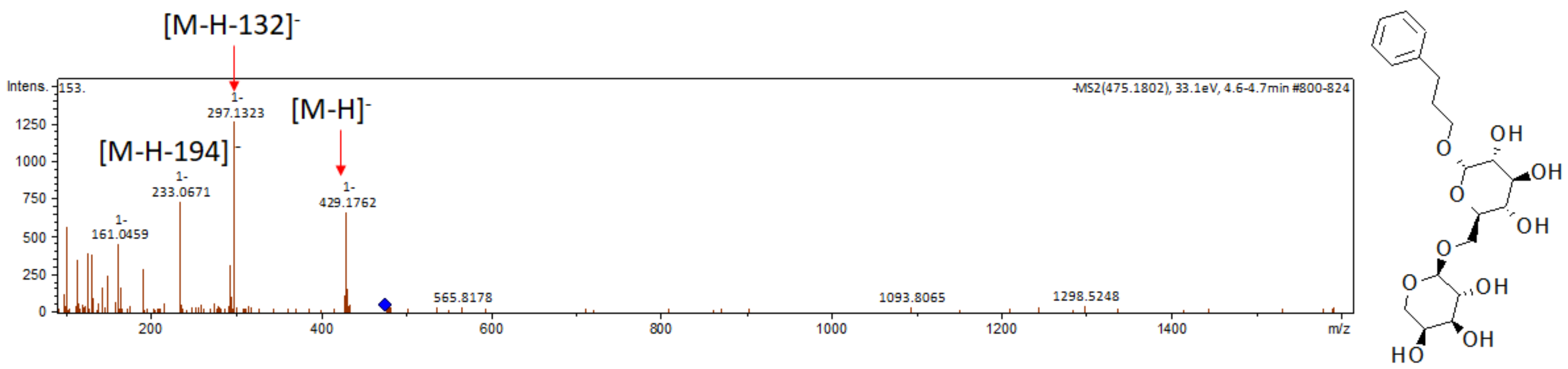

Figure S5. MS[2] Spectra of dihydrocinnamyl-*O*-pentosylhexoside (peak **27**) [M-H]- *m/z* 429.1762, $C_{20}H_{32}O_{12}$

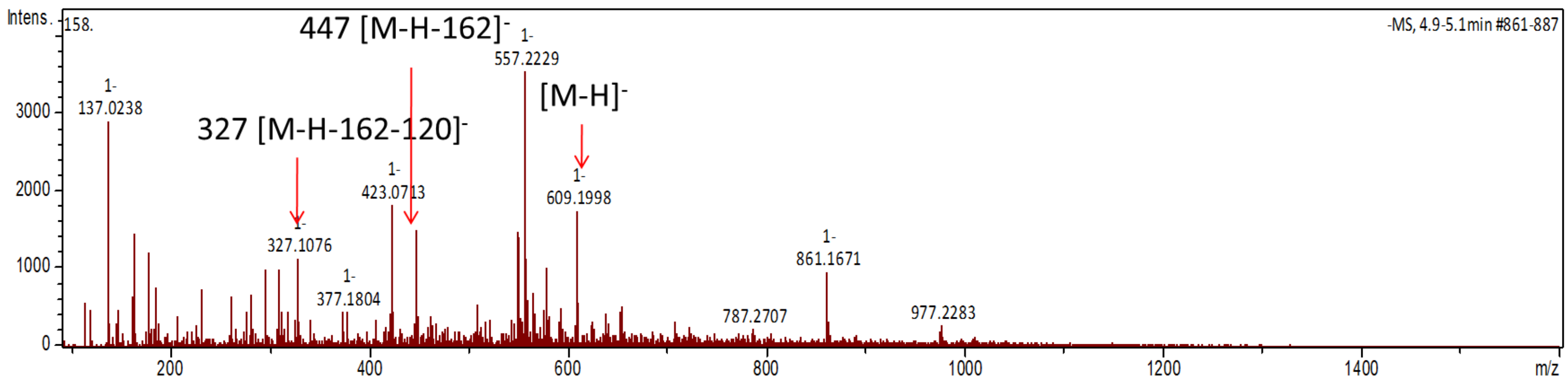

Figure S6. MS[2] Spectra of luteolin-*O/C*-di-hexoside (peak **30**) [M-H]- *m/z* 609.1998, $C_{27}H_{30}O_{16}$

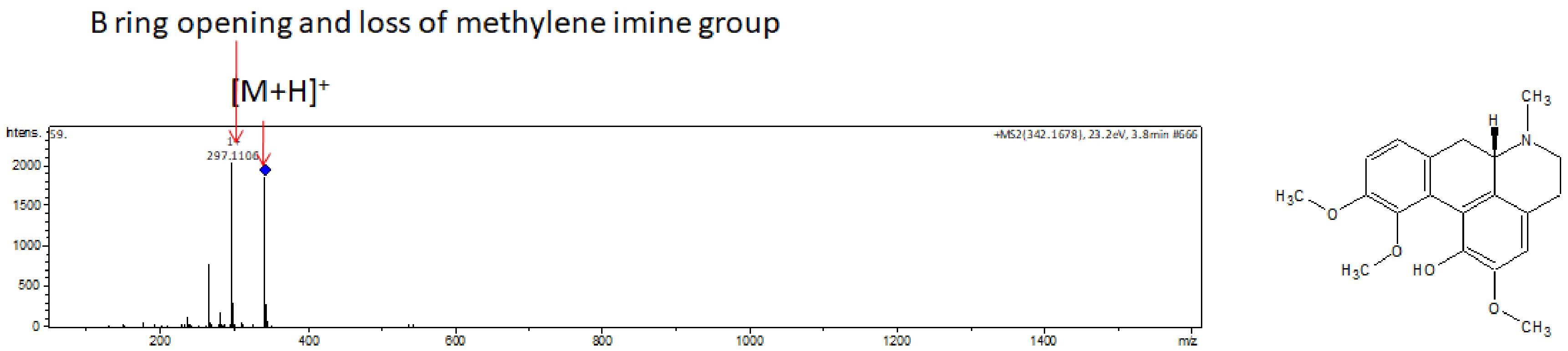

Figure S7. MS^2 Spectra of corydine (peak **21**) $[M+H]^+$ *m/z* 342.1678, $C_{20}H_{23}NO_4$

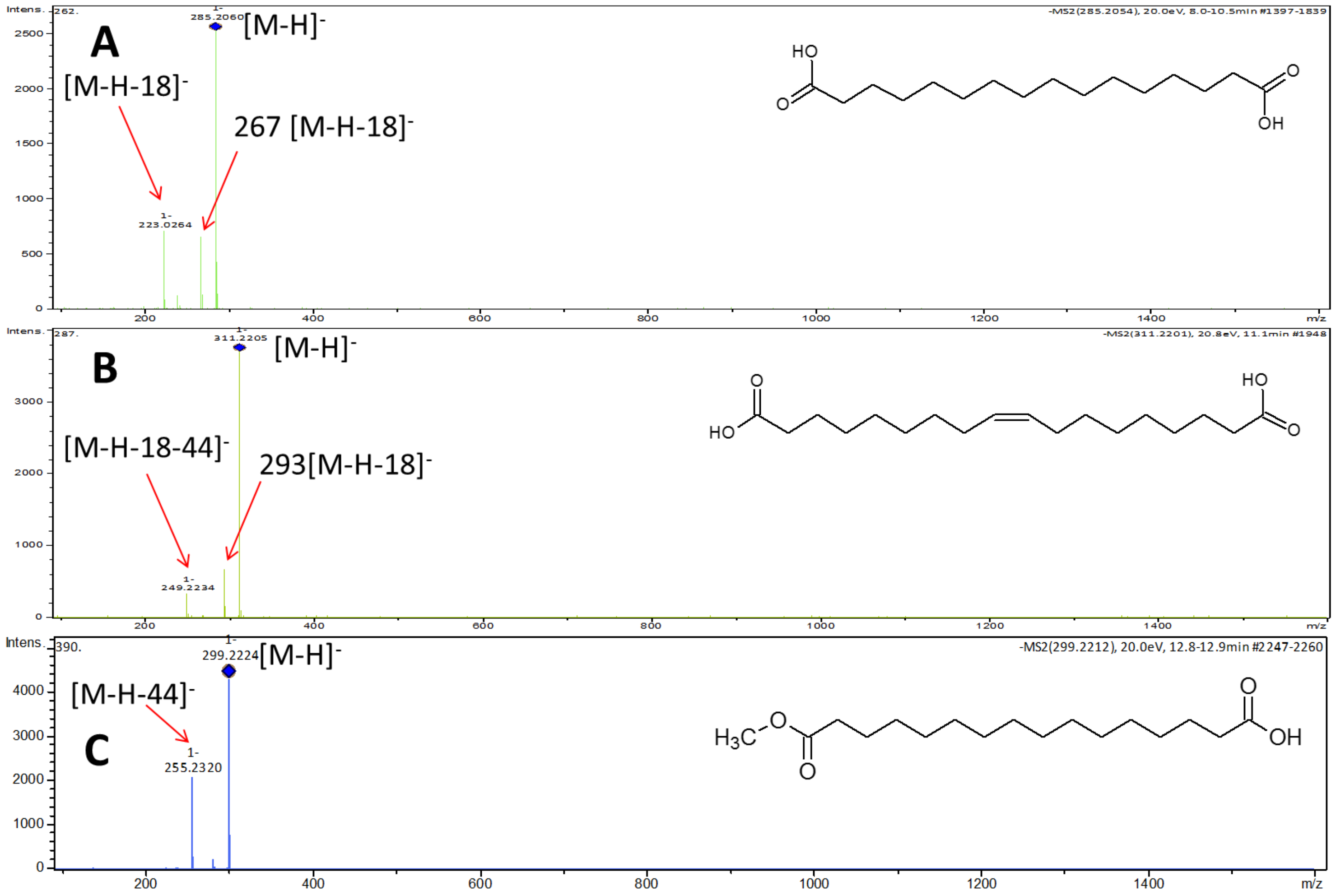

Figure S8. MS[2] Spectra of dicarboxylic fatty acids: **A** hexadecanedioic acid (peak **53**) [M-H]- *m/z* 285.2060,$C_{16}H_{31}O_4$; **B** octadecenedioic acid (peak **54**) [M-H]- *m/z* 311.2205, $C_{18}H_{32}O_4$; **C** Hexadecanedioic acid, monomethyl ester (peak **57**) [M-H]- *m/z* 299.2060, $C_{17}H_{32}O_4$

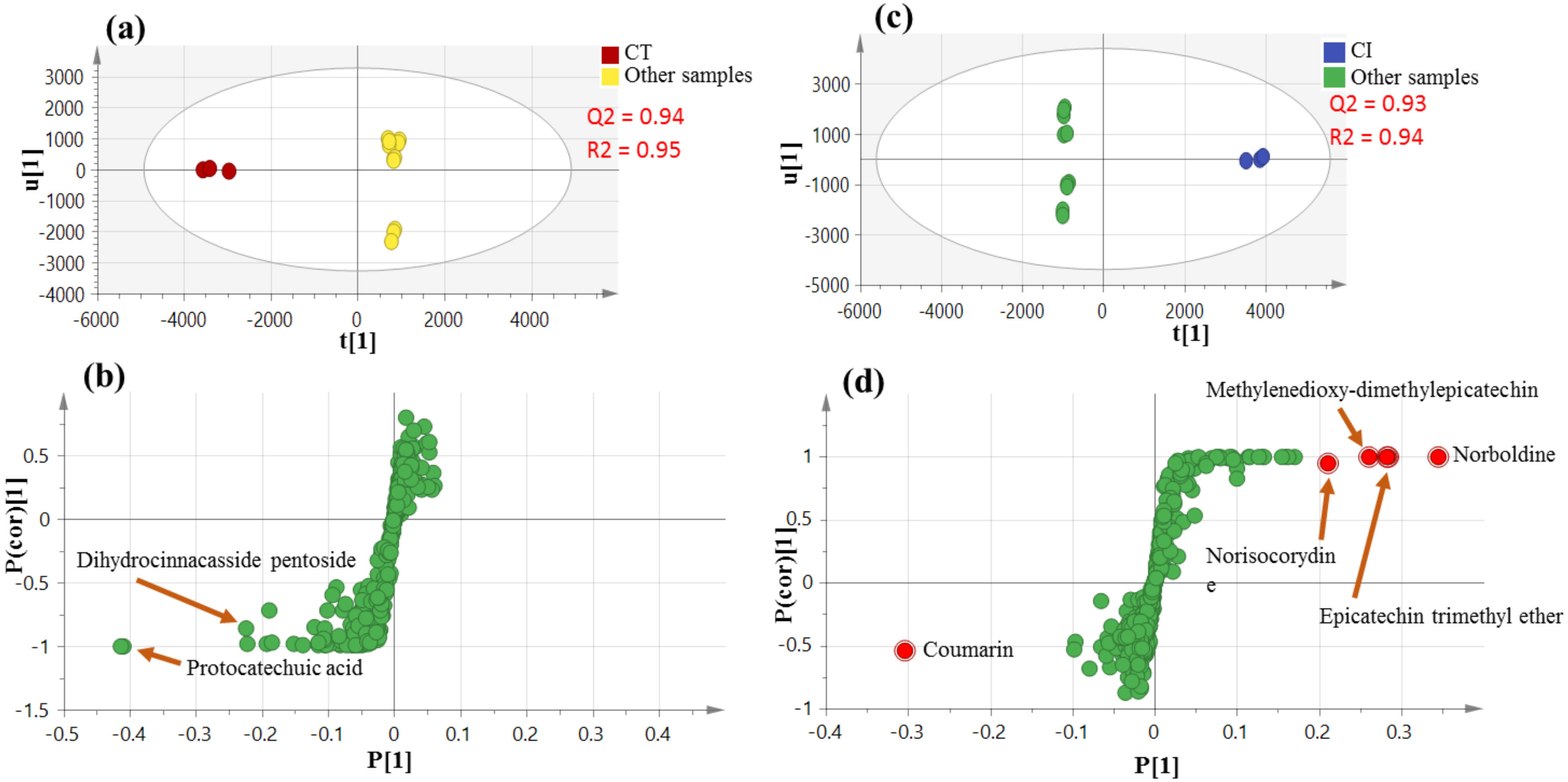

Figure S9. UPLC-MS OPLS-DA (a) score plot and (b) loading S-plots derived from modelling CT (*C. tamala* from Pakistan) against other samples in a separate group on negative ion mode. OPLS-DA (c) score plot and (d) loading S-plots derived from modelling CI (*C. iners* from Malaysia) against other samples in a separate group on positive ion mode.

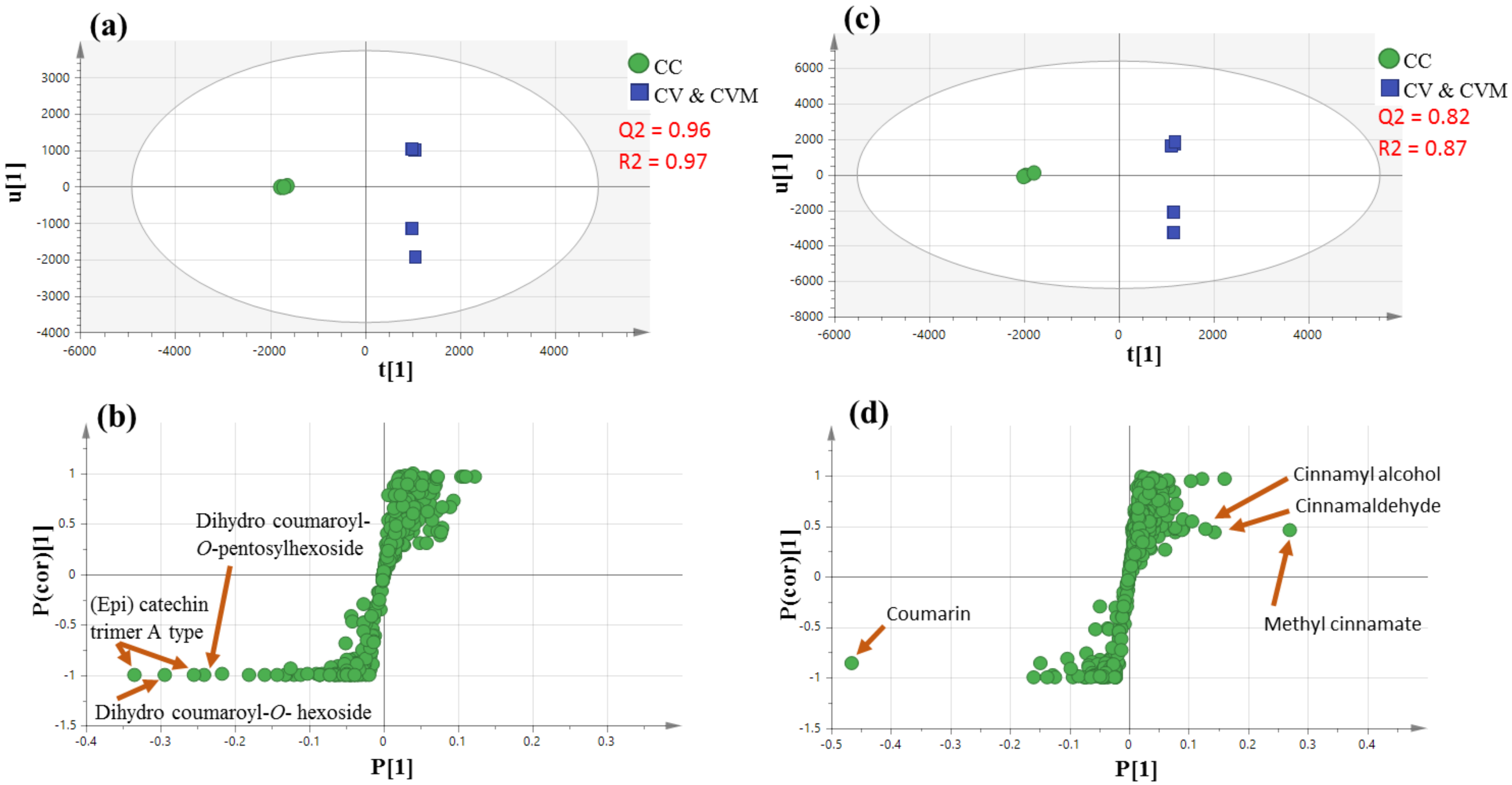

Figure S10. UPLC-MS OPLS-DA (a) score plot and (b) loading S-plots derived from modelling CC (*Cinnamomum cassia* from Malaysia) versus CV (*C. verum* from Pakistan) and CVM (*C. verum* from Malaysia) in a separate group on negative ion mode. OPLS-DA (c) score plot and (d) loading S-plots derived from modelling CC versus CV and CVM on positive ion mode. Each S-plot revealed the covariance p[1] against the correlation p(cor)[1] of the variables of the discriminating component.

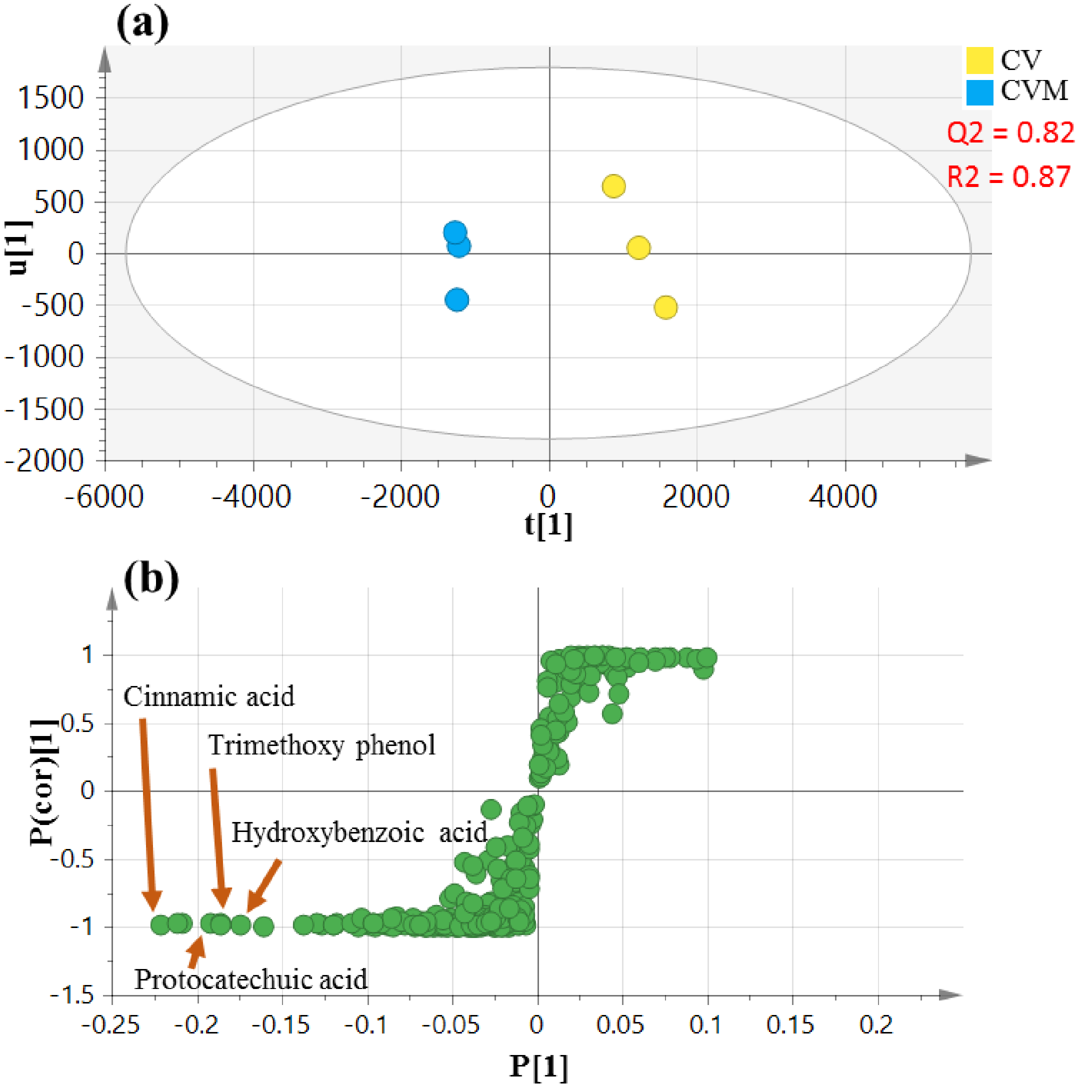

Figure S11. UPLC-MS OPLS-DA (a) score plot and (b) loading S-plots derived from modelling CV (*C. verum* from Pakistan) versus CVM (*C. verum* from Malaysia) on negative ion mode revealing the covariance p[1] against the correlation p(cor)[1] of the variables of the discriminating component.

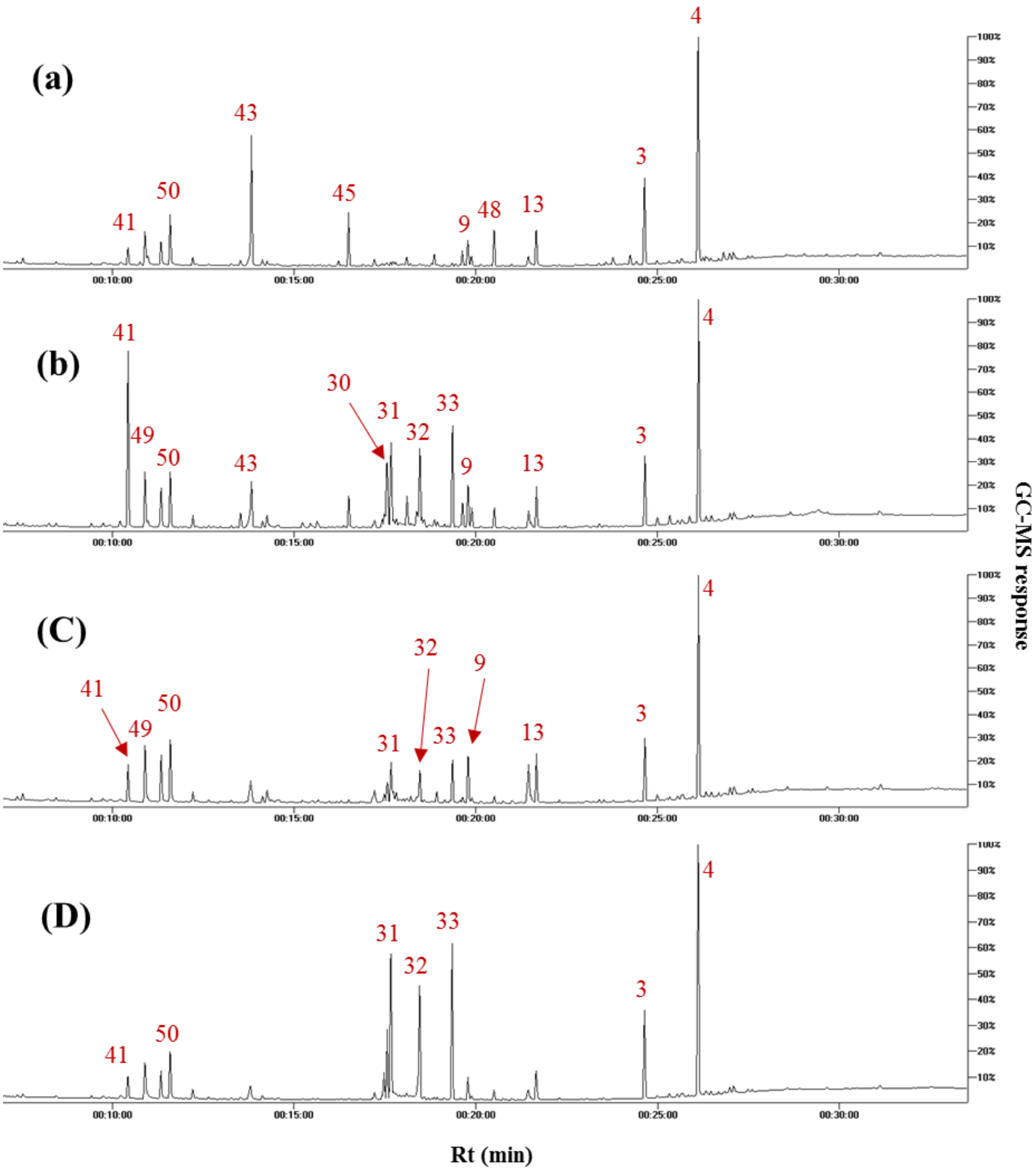

Figure S12. Representative SPME-GC-MS chromatograms of cinnamon primary metabolites, acquired from (a) CI (*C. iners* from Malaysia), (b) CT (*C. tamala* from Pakistan) and (c) CV (*C. verum* from Pakistan).

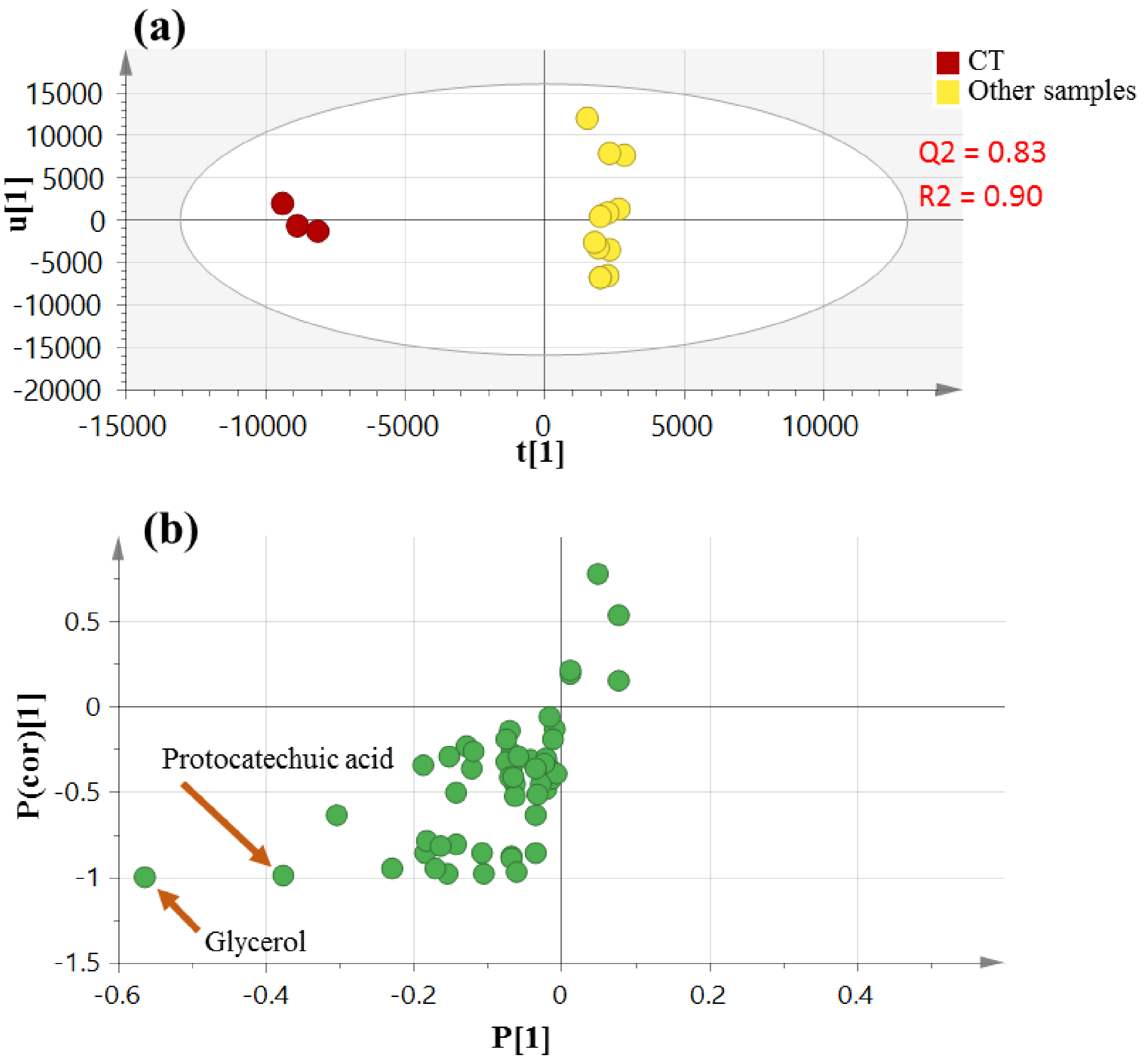

Figure S13. GC-MS OPLS-DA (a) score plot and (b) loading S-plots derived from modelling CT (*C. tamala* from Pakistan) versus all other samples revealing the covariance p[1] against the correlation p(cor)[1] of the variables of the discriminating component.

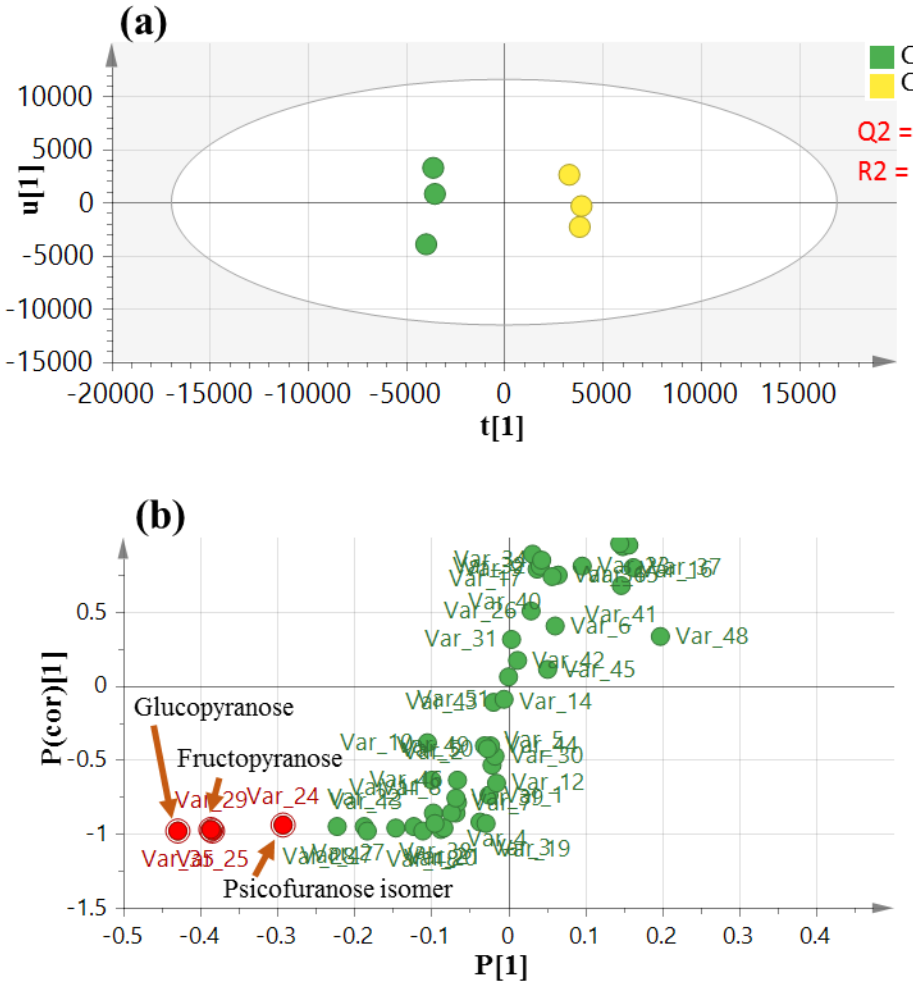

Figure S14. GC-MS OPLS-DA (a) score plot and (b) loading S-plots derived from modelling CC (*Cinnamomum cassia* from Malaysia) versus CV (*C. verum* from Pakistan) revealing the covariance p[1] against the correlation p(cor)[1] of the variables of the discriminating component.
